# Supplementary material for: Atomically dispersed Pt–N4 sites as efficient and selective electrocatalysts for the chlorine evolution reaction
Source: Nat Commun. 2020 Jan 21;11:412. doi: 10.1038/s41467-019-14272-1 (PMC6972710; doi:10.1038/s41467-019-14272-1)
Supplement: Supplementary file 1 — Supplementary Information [file 41467_2019_14272_MOESM1_ESM.pdf]

Supplementary Information

Atomically dispersed Pt–N_4 sites as efficient and selective electrocatalysts for the chlorine evolution reaction

Taejung Lim,[1,4] Gwan Yeong Jung,[1,4] Jae Hyung Kim,[1] Sung O Park,[1] Jaehyun Park,[1] Yong-Tae Kim,[2] Seok Ju Kang,[1] Hu Young Jeong,[3] Sang Kyu Kwak,[*,1] and Sang Hoon Joo[*,1]

[1]Department of Energy Engineering and School of Energy and Chemical Engineering, Ulsan National Institute of Science and Technology (UNIST), 50 UNIST-gil, Ulsan 44919, Republic of Korea.

[2]Department of Materials Science and Engineering, Pohang University of Science and Technology (POSTECH), 77 Cheongam-Ro, Pohang, Gyeongbuk 37673, Republic of Korea

[3]UNIST Central Research Facilities, Ulsan National Institute of Science and Technology (UNIST), 50 UNIST-gil, Ulsan 44919, Republic of Korea.

[4]These authors contributed equally: Taejung Lim, Gwan Yeong Jung

* Correspondence and requests for materials should be addressed to S.H.J. (shjoo@unist.ac.kr) or to S.K.K. (skkwak@unist.ac.kr).

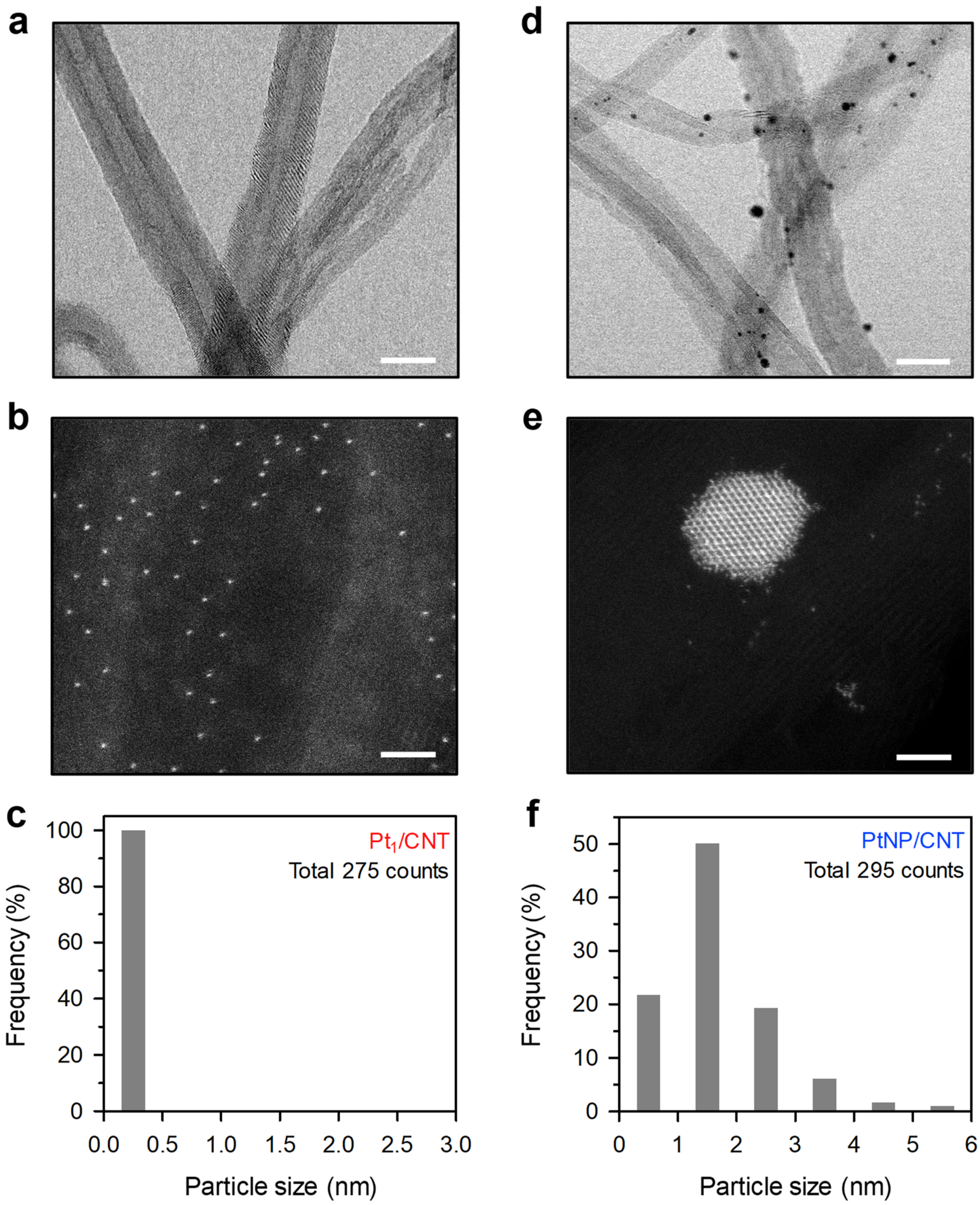

Supplementary Fig. 1 | Additional TEM images and Pt particle size distribution histograms of Pt_1/CNT and PtNP/CNT catalysts. **a** Low-magnification HR-TEM image, **b** HAADF-STEM image, and **c** Pt particles size distribution histogram of Pt_1/CNT catalyst. **d** Low-magnification HR-TEM image, **e** HAADF-STEM image, and **f** Pt particles size distribution histogram of PtNP/CNT catalyst. Scale bars: 20 nm in **a**, **d** and 2 nm in **b**, **e**.

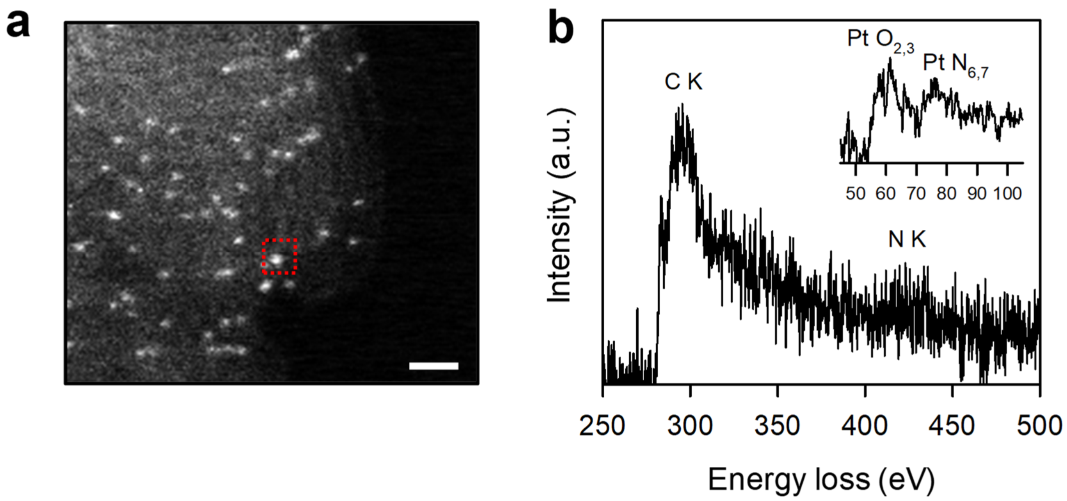

Supplementary Fig. 2 | EELS spectrum of Pt_1/CNT. **a** High-magnification HAADF-STEM image. Scale bar: 1 nm. **b** EELS spectrum of Pt_1/CNT taken on the red dotted box (~5 $Å^2$) in **a**.

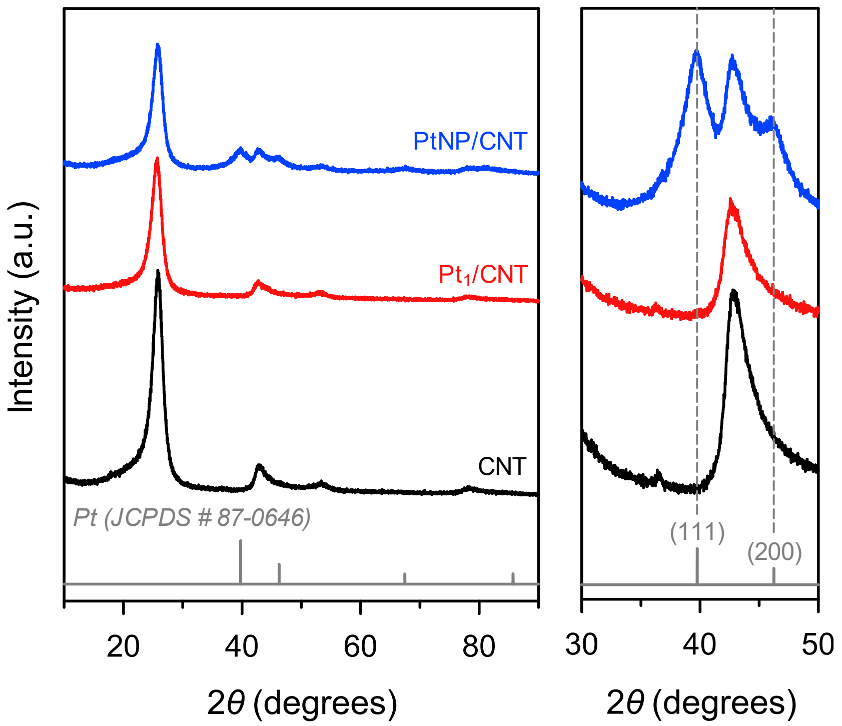

Supplementary Fig. 3 | XRD patterns of CNT, Pt_1/CNT, and PtNP/CNT catalysts. Reference for the face-centred cubic (fcc) Pt (JCPDS # 87-0646) is also shown.

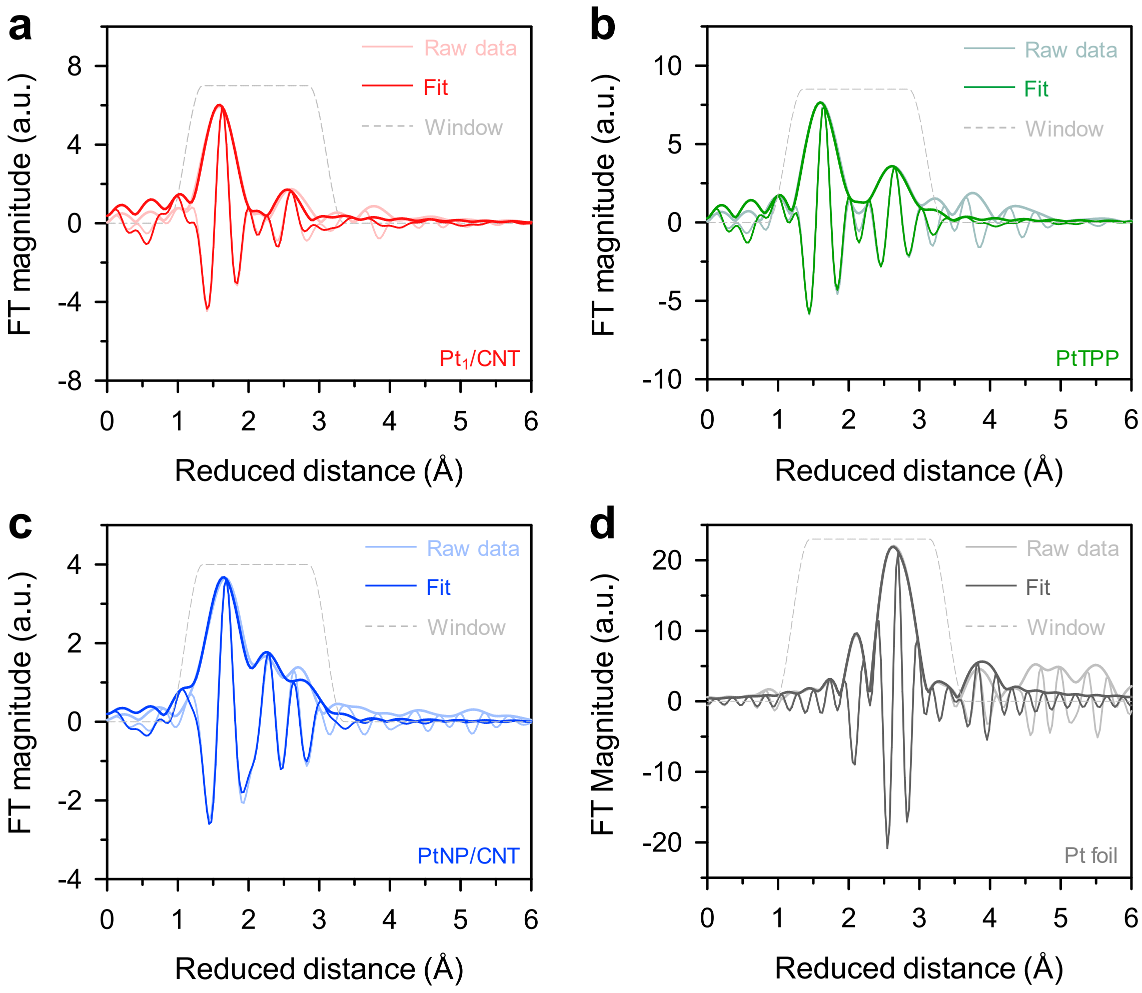

Supplementary Fig. 4 | k^3-weighted Pt L_3-edge EXAFS spectra and fitted curves of Pt_1/CNT catalyst, PtTPP precursor, PtNP/CNT catalyst, and Pt foil. **a** Pt_1/CNT catalyst, **b** PtTPP precursor, **c** PtNP/CNT catalyst, and **d** Pt foil.

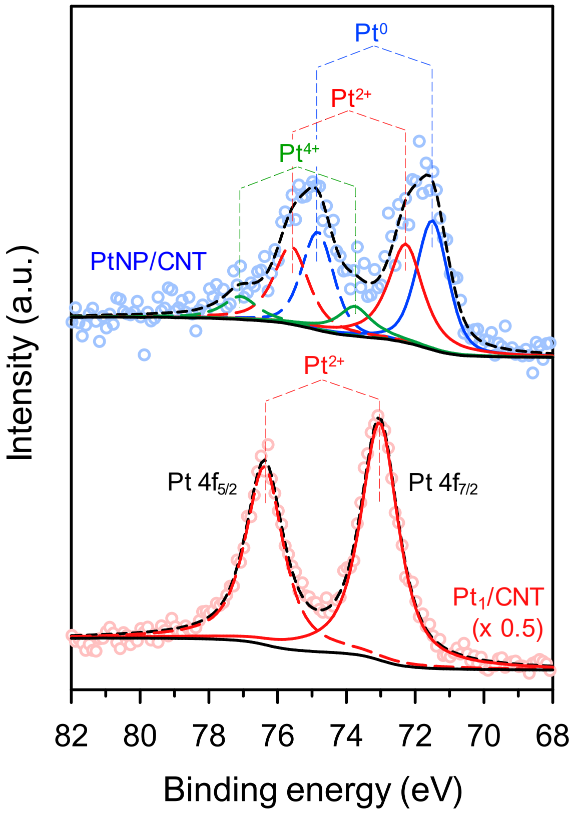

Supplementary Fig. 5 | Deconvoluted Pt 4f XPS spectra of Pt_1/CNT and PtNP/CNT catalysts. The spin-orbit splitting and area ratio for $4f_{5/2}$ (dashed lines) and $4f_{7/2}$ (solid lines) peaks are 3.34 eV and 3:4, respectively. The peak of Pt $4f_{7/2}$ in the spectrum of Pt_1/CNT catalyst was observed at 73.1 eV, which is close to the value of Pt porphyrin in a previous report[1].

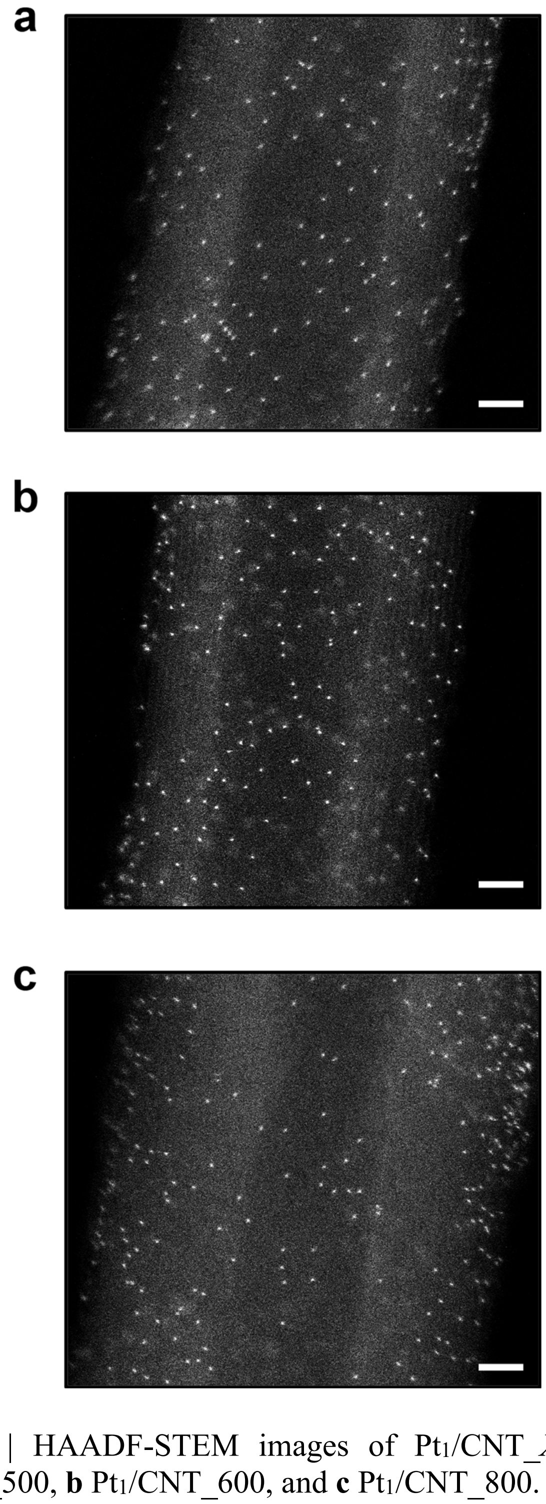

Supplementary Fig. 6 | HAADF-STEM images of Pt_1/CNT_*X* catalysts (*X*=annealing temperature). **a** Pt_1/CNT_500, **b** Pt_1/CNT_600, and **c** Pt_1/CNT_800. Scale bars: 2 nm.

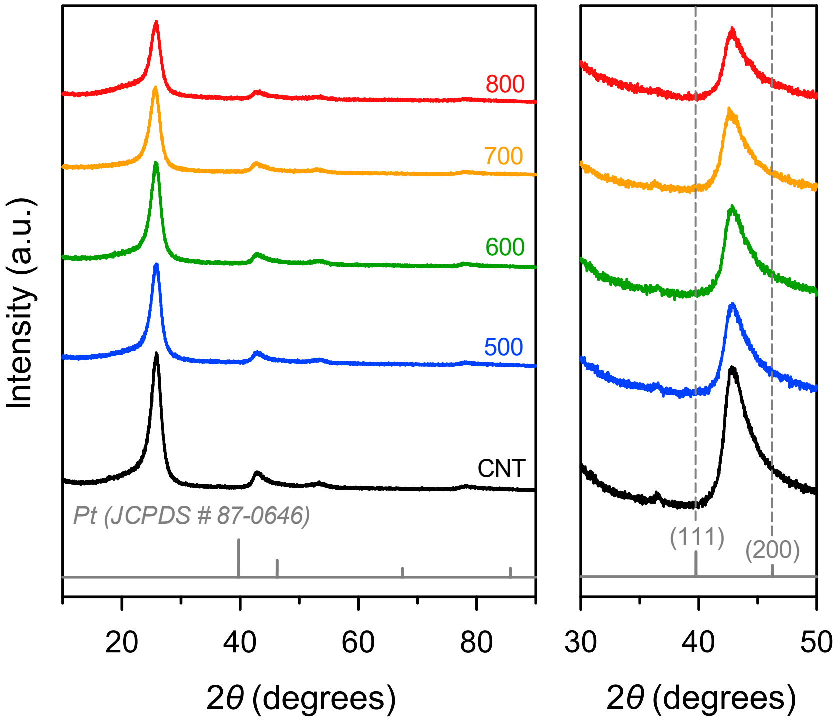

Supplementary Fig. 7 | XRD patterns of CNT and Pt_1/CNT_*X* catalysts. Reference for the face-centred cubic (fcc) Pt (JCPDS # 87-0646) is also shown.

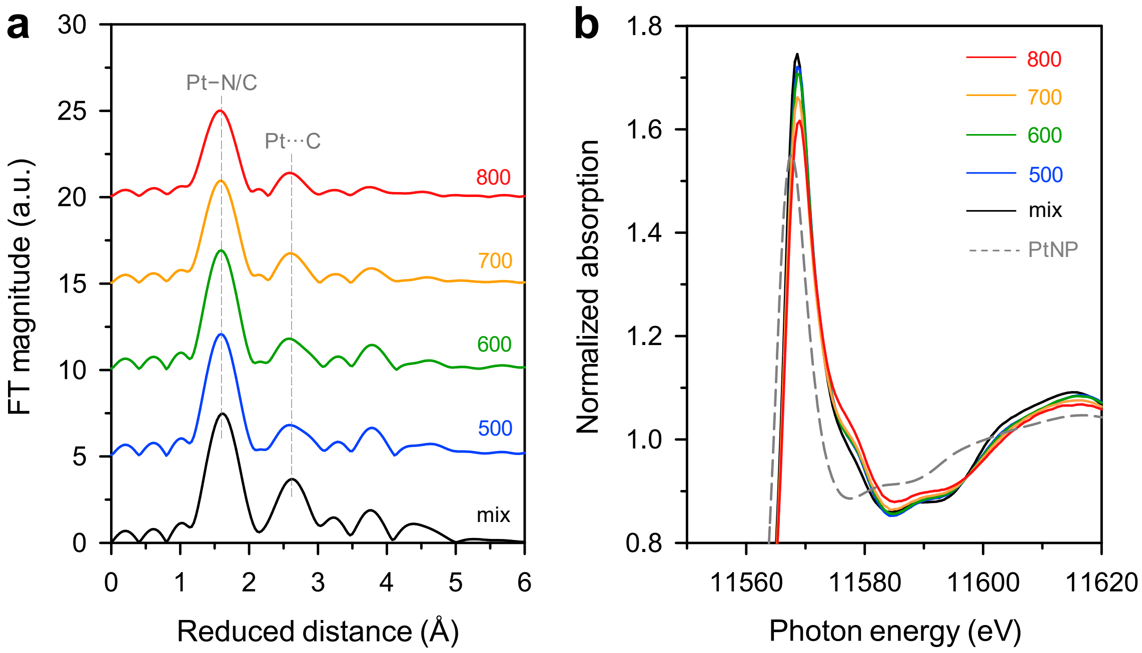

Supplementary Fig. 8 | k^3-weighted Pt L$_3$-edge EXAFS and XANES spectra of Pt$_1$/CNT_X catalysts and unpyrolysed mixture of PtTPP precursor and CNT. **a** EXAFS spectra. **b** Enlarged white line region of XANES spectra. Unpyrolysed mixture of PtTPP and CNT was denoted as "mix". The XANES spectrum of PtNP/CNT catalyst was also displayed in **b** for comparison.

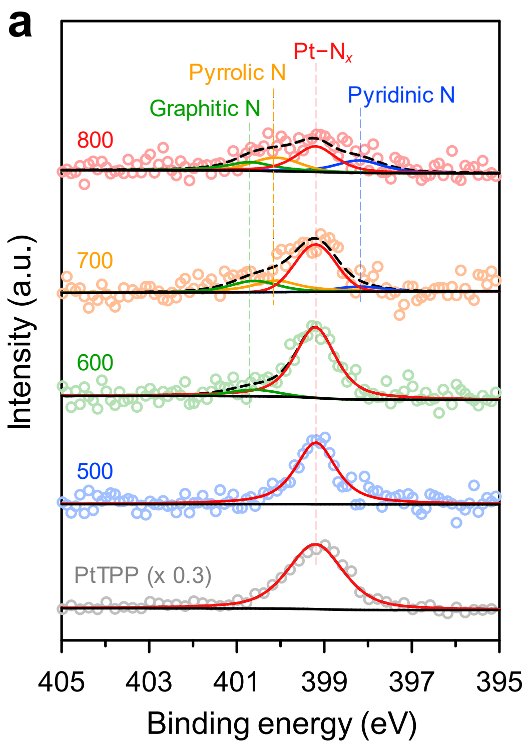

Supplementary Fig. 9 | Deconvoluted N 1s and Pt 4f XPS spectra of Pt_1/CNT_*X* catalysts and PtTPP precursor. **a** Deconvoluted N 1s XPS spectra. **b** Deconvoluted Pt 4f XPS spectra. In **b**, the spin-orbit splitting and area ratio for $4f_{5/2}$ (dashed lines) and $4f_{7/2}$ (solid lines) peaks are 3.34 eV and 3:4, respectively.

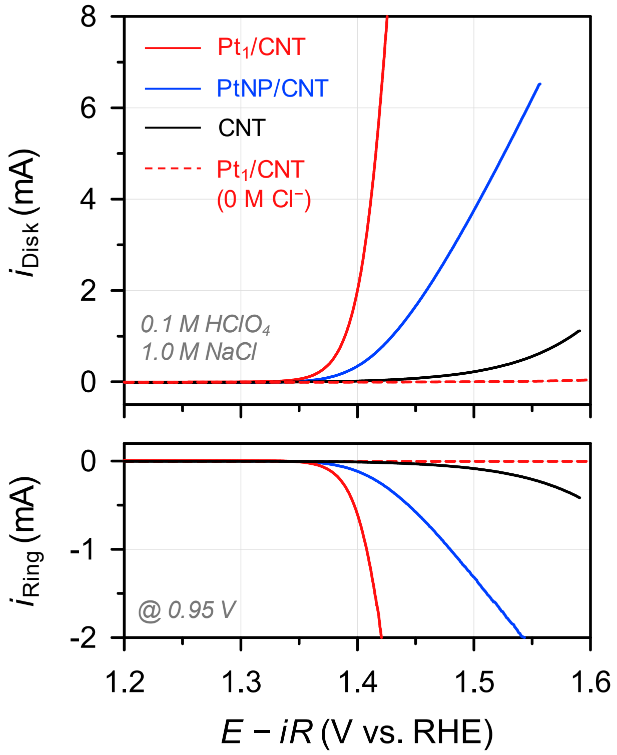

Supplementary Fig. 10 | RRDE measurements of CER activities on Pt_1/CNT, PtNP/CNT catalysts, and CNT in acidic media with 1.0 M NaCl. The measurement conditions were Ar-saturated 0.1 M $HClO_4$, an electrode rotation speed of 1600 rpm, and a scan rate of 10 mV s^{-1}. Top panel indicates disk currents for CER. Bottom panel shows the corresponding ring current for Cl_2 reduction obtained on Pt ring electrode, whose potential was fixed at 0.95 V.

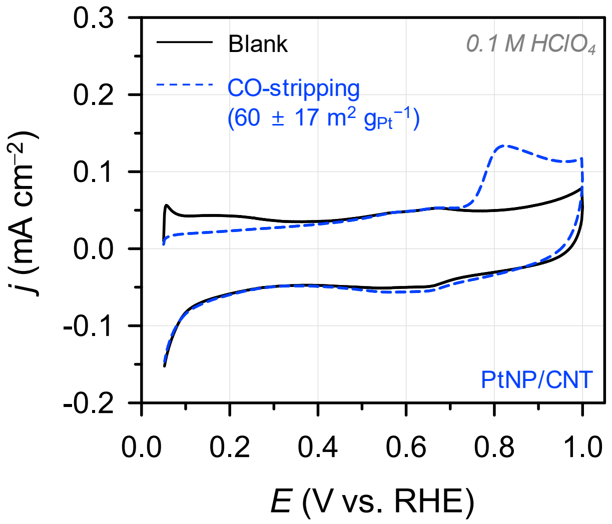

Supplementary Fig. 11 | Electrochemical CO stripping results of PtNP/CNT catalyst in acidic media. The calculated electrochemically active surface area of PtNP/CNT is noted in parenthesis.

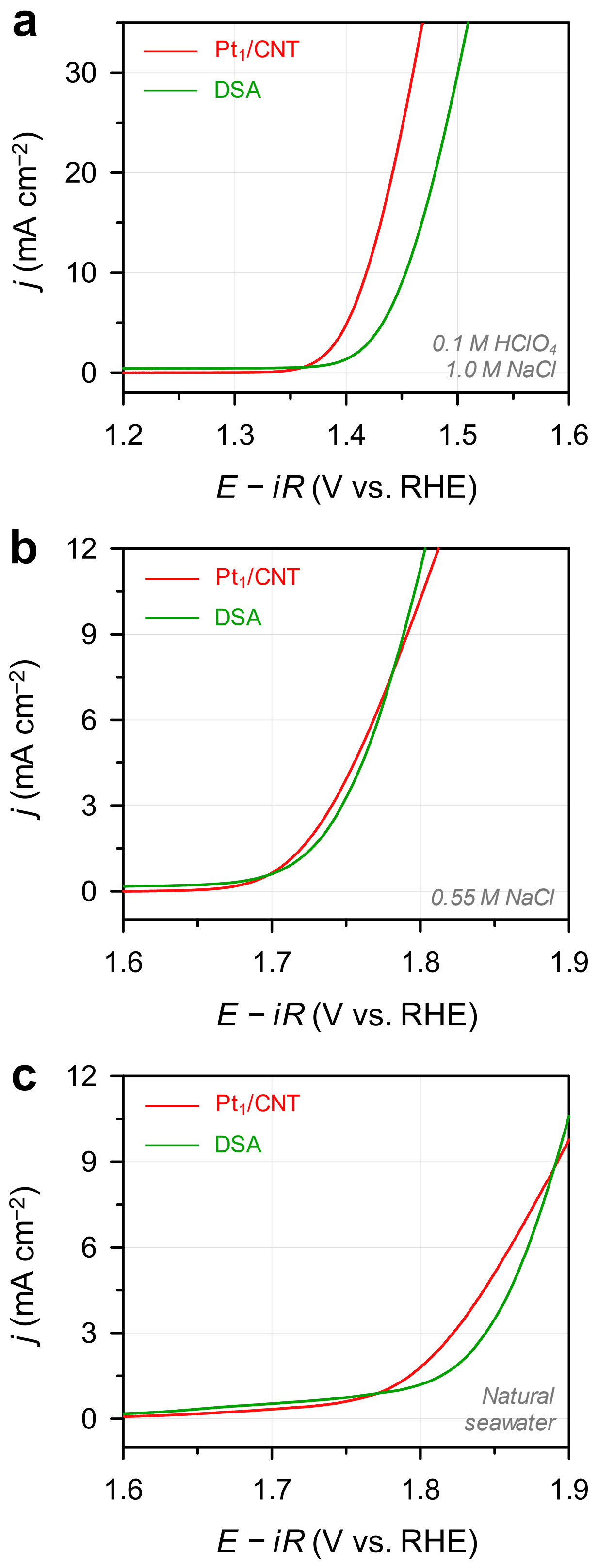

Supplementary Fig. 12 | CER polarisation curves of Pt_1/CNT catalyst on a carbon paper and DSA in different electrolyte conditions. **a** 0.1 M $HClO_4$ + 1.0 M NaCl, **b** 0.55 M NaCl, and **c** natural seawater. A scan rate of 10 mV s^{-1} were used.

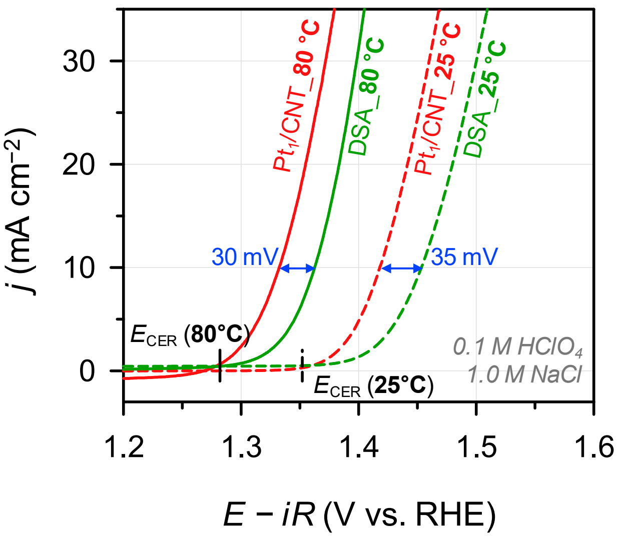

Supplementary Fig. 13 | CER polarisation curves of Pt_1/CNT catalyst loaded on a carbon paper and DSA in 0.1 M $HClO_4$ + 1.0 M NaCl at 80 °C. The equilibrium potential of CER (E_{CER}) depends on the temperature (see **Methods** in the manuscript).

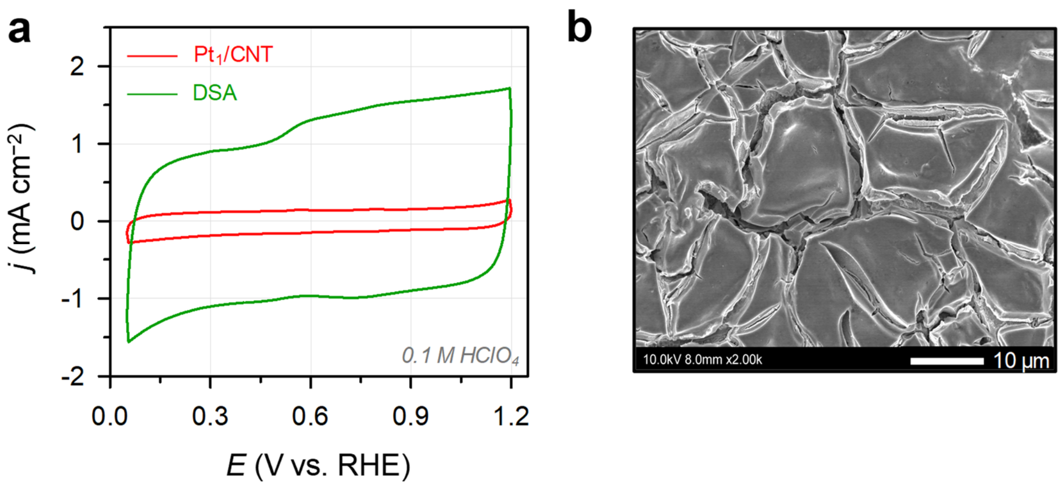

Supplementary Fig. 14 | Cyclic voltammograms of Pt_1/CNT catalyst on a carbon paper and DSA in acidic media with 1.0 M NaCl and SEM image of DSA. **a** Cyclic voltammograms in Ar-saturated 0.1 M $HClO_4$ at a scan rate of 50 mV s^{-1}. **b** SEM image of DSA.

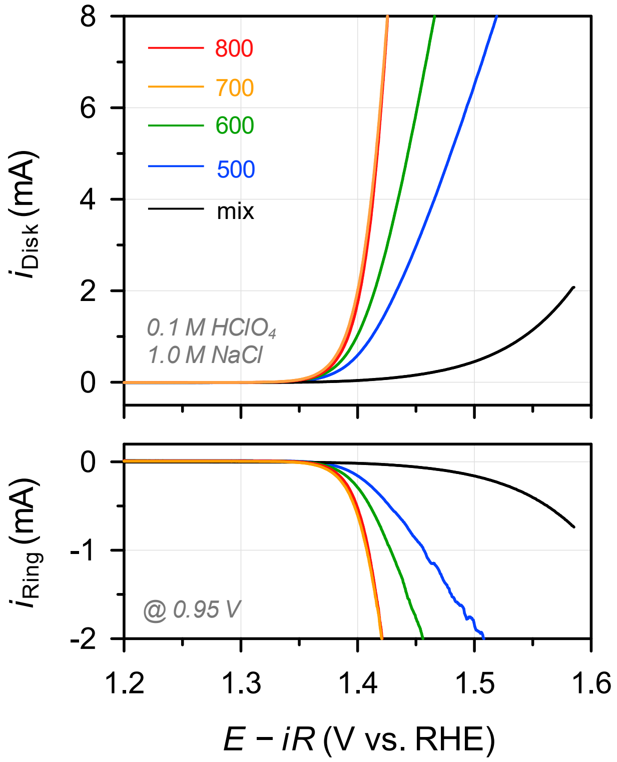

Supplementary Fig. 15 | RRDE measurements of CER activities on Pt_1/CNT_*X* catalysts and unpyrolysed mixture of PtTPP precursor and CNT in acidic media with 1.0 M NaCl. The measurement conditions were Ar-saturated 0.1 M $HClO_4$, an electrode rotation speed of 1600 rpm, and a scan rate of 10 mV s^{-1}. Top panel indicates disk currents for CER. Bottom panel shows the corresponding ring current for Cl_2 reduction obtained on Pt ring electrode, whose potential was fixed at 0.95 V.

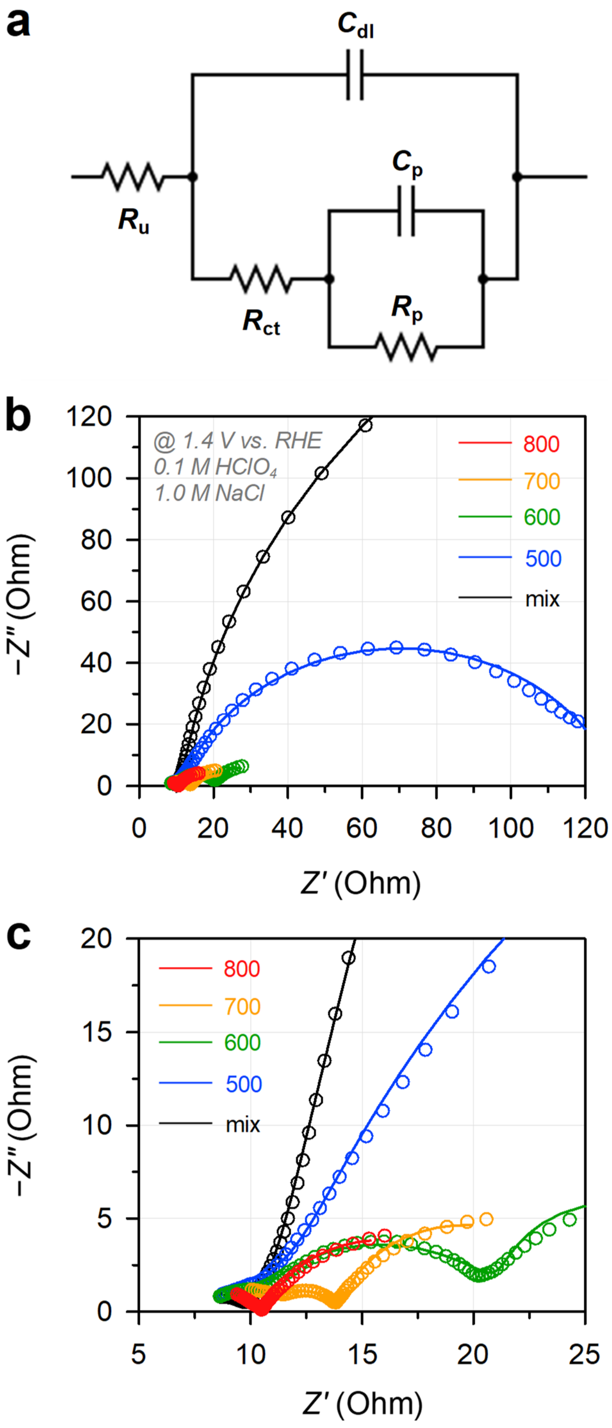

Supplementary Fig. 16 | Nyquist plots and corresponding fitting results of Pt_1/CNT_*X* catalysts and unpyrolysed mixture of PtTPP precursor and CNT in acidic media with 1.0 M NaCl. **a** Equivalent circuit for EIS fitting. **b** Nyquist plots with high impedance range. **c** Nyquist plots with low impedance range. The measurement conditions were Ar-saturated 0.1 M $HClO_4$ + 1.0 M NaCl, a fixed potential of 1.4 V, an electrode rotation speed of 1600 rpm, and a frequency range of 1−100,000 Hz. The empty circle and solid line in the Nyquist plots indicate experimental and fitting results, respectively.

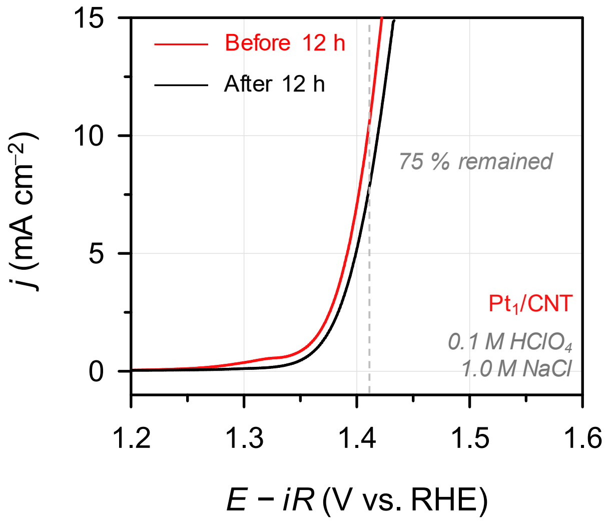

Supplementary Fig. 17 | CER polarisation curves of Pt_1/CNT catalyst loaded on a carbon paper before and after 12 h of stability test at 10 mA cm^{-2} at a scan rate of 10 mV s^{-1} (**Fig. 1c**). An electrolyte was stirred at a rotation speed of 300 rpm. The electrolyte was replaced with a fresh electrolyte after the stability test.

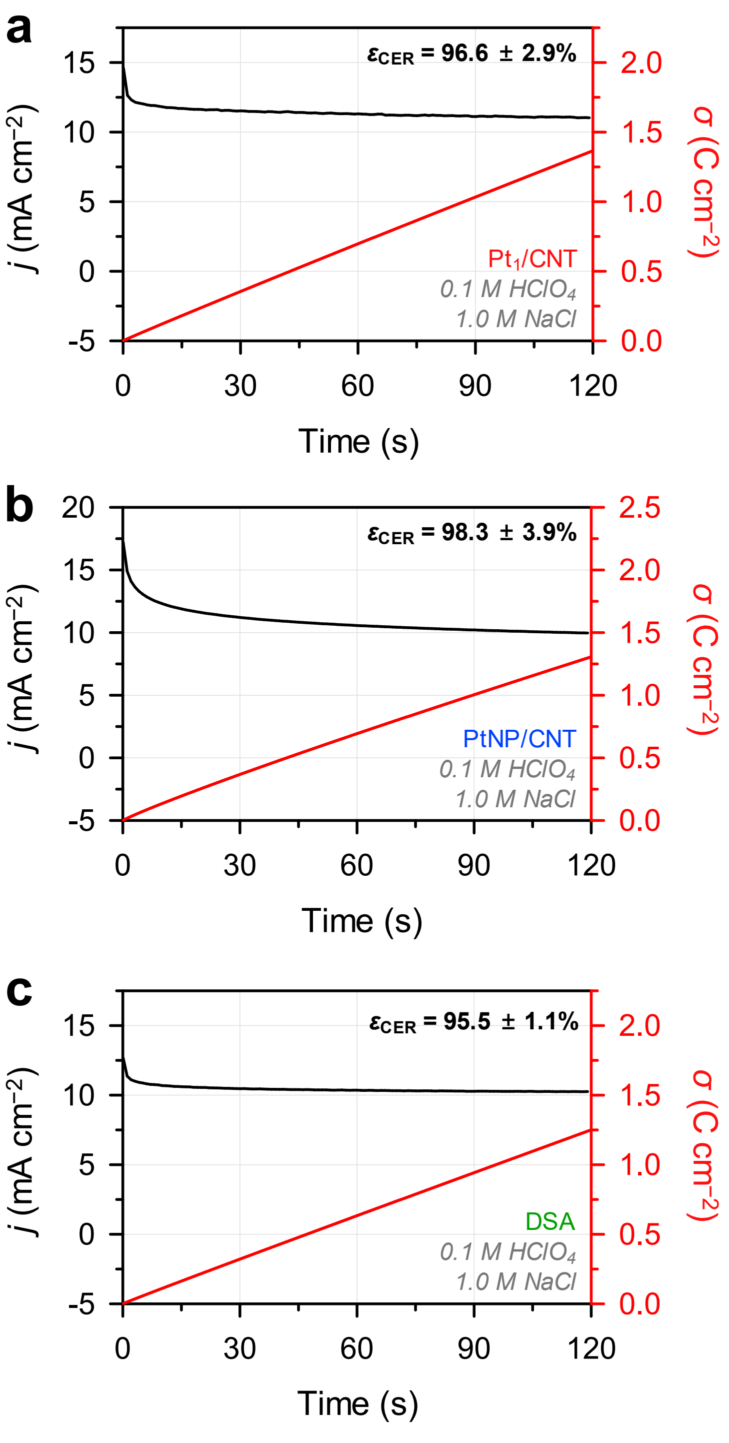

Supplementary Fig. 18 | Chronoamperograms for iodometric titration of Cl_2 product on Pt_1/CNT, PtNP/CNT catalysts, and DSA in acidic media with 1.0 M NaCl. **a** Pt_1/CNT, **b** PtNP/CNT, and **c** DSA catalysts. The electrolytes were Ar-saturated.

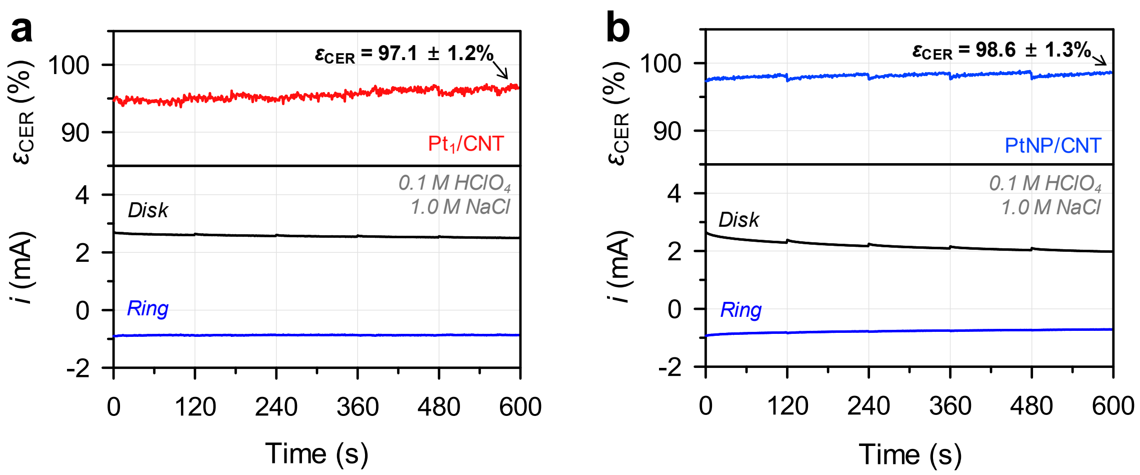

Supplementary Fig. 19 | Chronoamperograms of Pt_1/CNT and PtNP/CNT catalysts measured by RRDE in acidic media with 1.0 M NaCl. **a** Pt_1/CNT and **b** PtNP/CNT catalysts. The measurement conditions were Ar-saturated 0.1 M $HClO_4$ and an electrode rotation speed of 1600 rpm. The potential of the Pt ring electrode was fixed at 0.95 V for Cl_2 reduction.

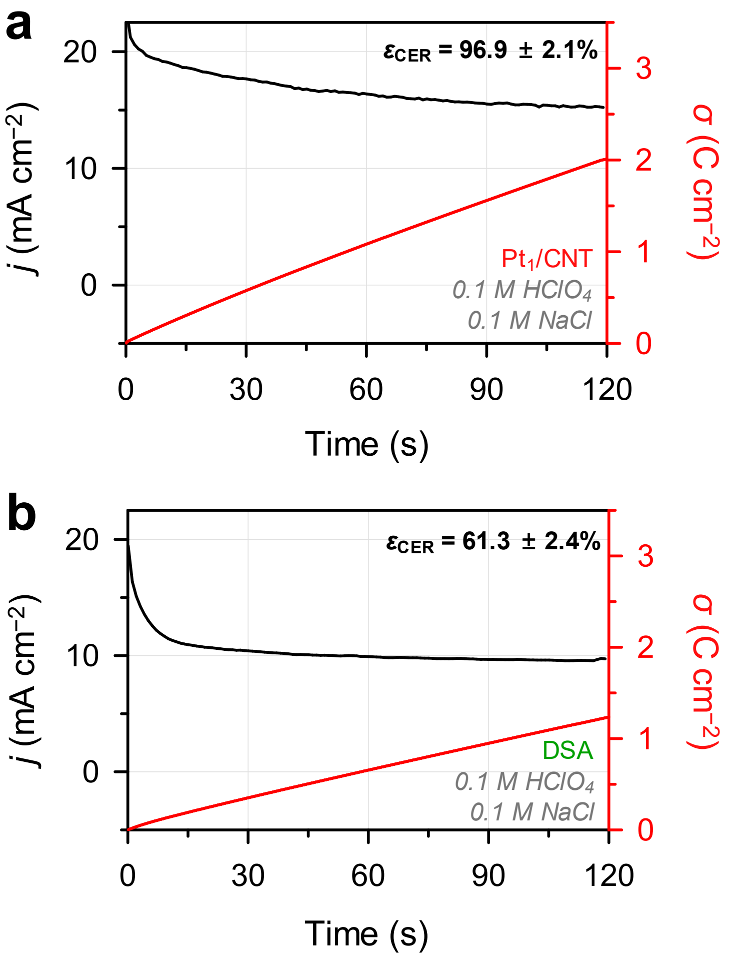

Supplementary Fig. 20 | Chronoamperograms for iodometric titration of Cl_2 product on Pt_1/CNT catalyst and DSA in acidic media with 0.1 M NaCl. **a** Pt_1/CNT and **b** DSA catalysts. The electrolytes were Ar-saturated.

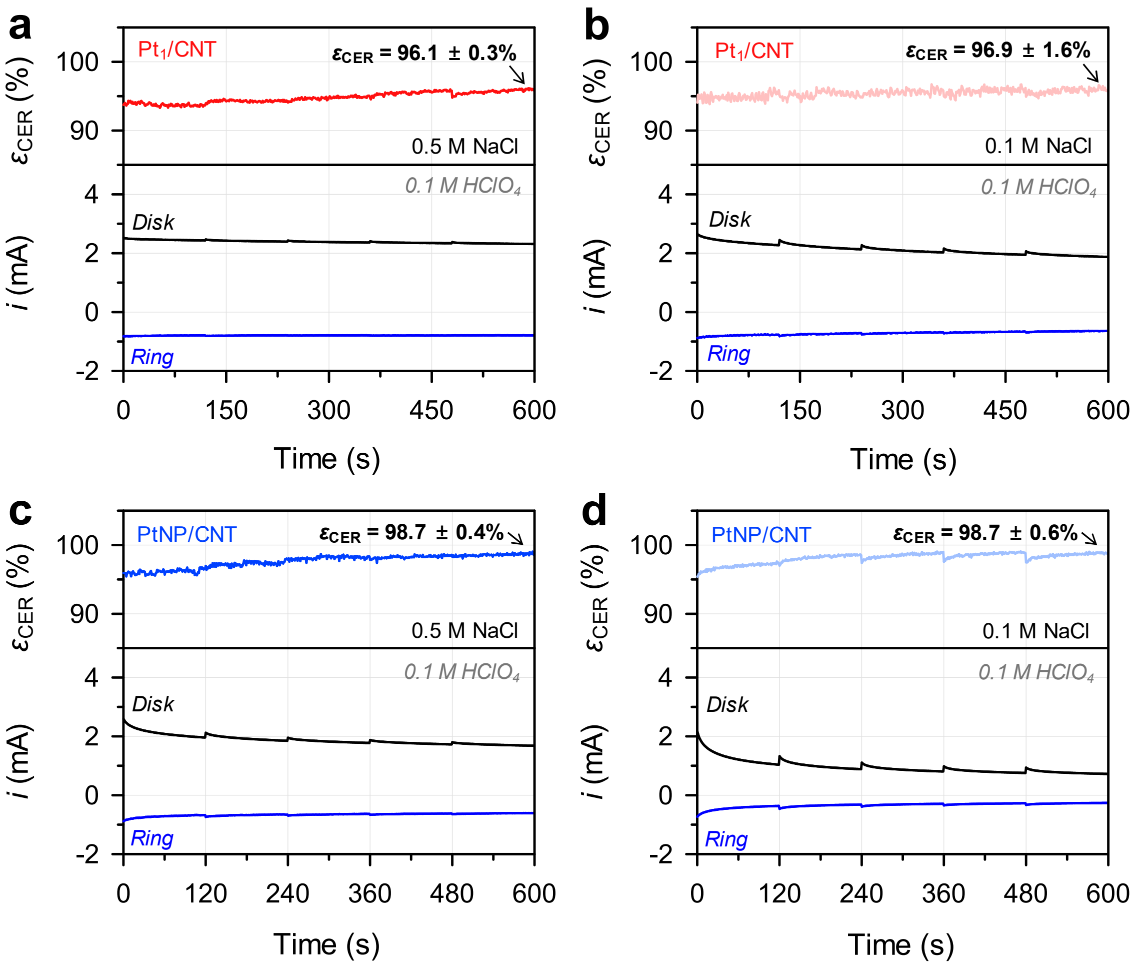

Supplementary Fig. 21 | Chronoamperograms of Pt_1/CNT and PtNP/CNT catalysts measured by RRDE in acidic media with 0.5 M or 0.1 M NaCl. The CER selectivity of Pt_1/CNT in **a** 0.5 M NaCl and **b** 0.1 M NaCl, and PtNP/CNT in **c** 0.5 M NaCl and **d** 0.1 M NaCl. The measurement conditions were Ar-saturated 0.1 M $HClO_4$ and an electrode rotation speed of 1600 rpm. The potential of the Pt ring electrode was fixed at 0.95 V for Cl_2 reduction.

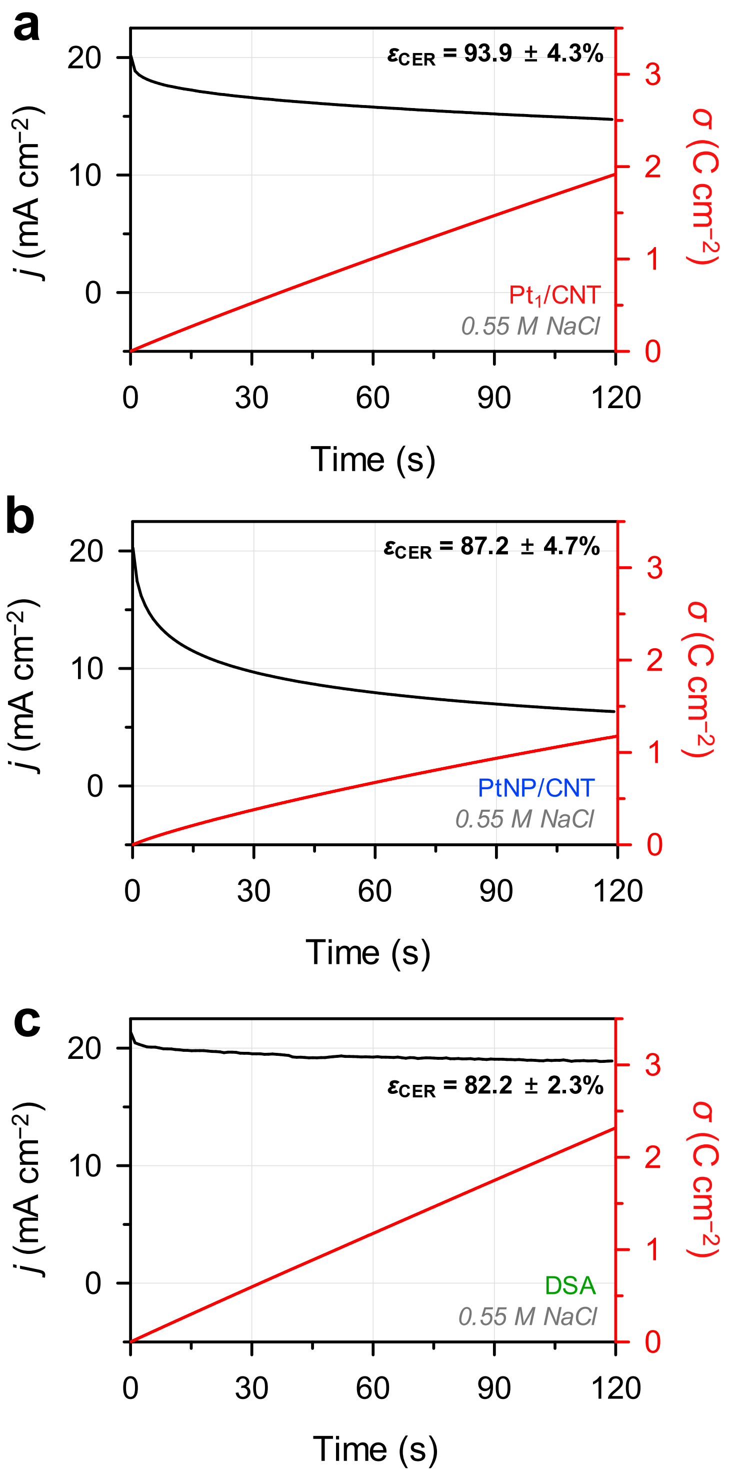

Supplementary Fig. 22 | Chronoamperograms for iodometric titration of Cl_2 product on Pt_1/CNT, PtNP/CNT, and DSA catalysts in neutral media with 0.55 M NaCl. **a** Pt_1/CNT, **b** PtNP/CNT, and **c** DSA catalysts. The electrolytes were Ar-saturated.

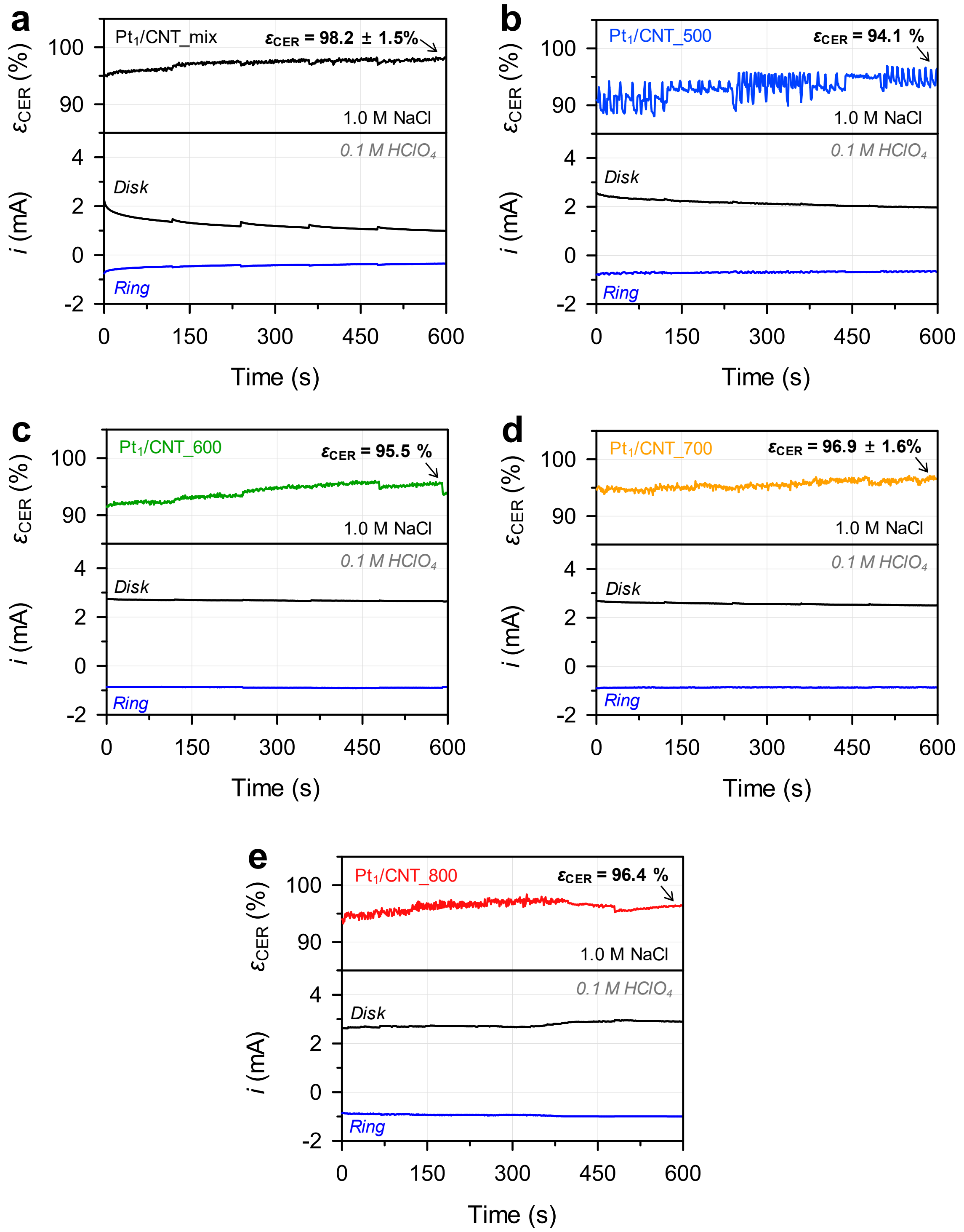

Supplementary Fig. 23 | Chronoamperograms of Pt_1/CNT_*X* catalysts measured by RRDE in acidic media with 1.0 M NaCl. **a** Pt_1/CNT_mix, **b** Pt_1/CNT_500, **c** Pt_1/CNT_600, **d** Pt_1/CNT_700, and **e** Pt_1/CNT_800. Measurement conditions were Ar-saturated 0.1 M $HClO_4$ and an electrode rotation speed of 1600 rpm. The potential of the Pt ring electrode was fixed at 0.95 V for Cl_2 reduction.

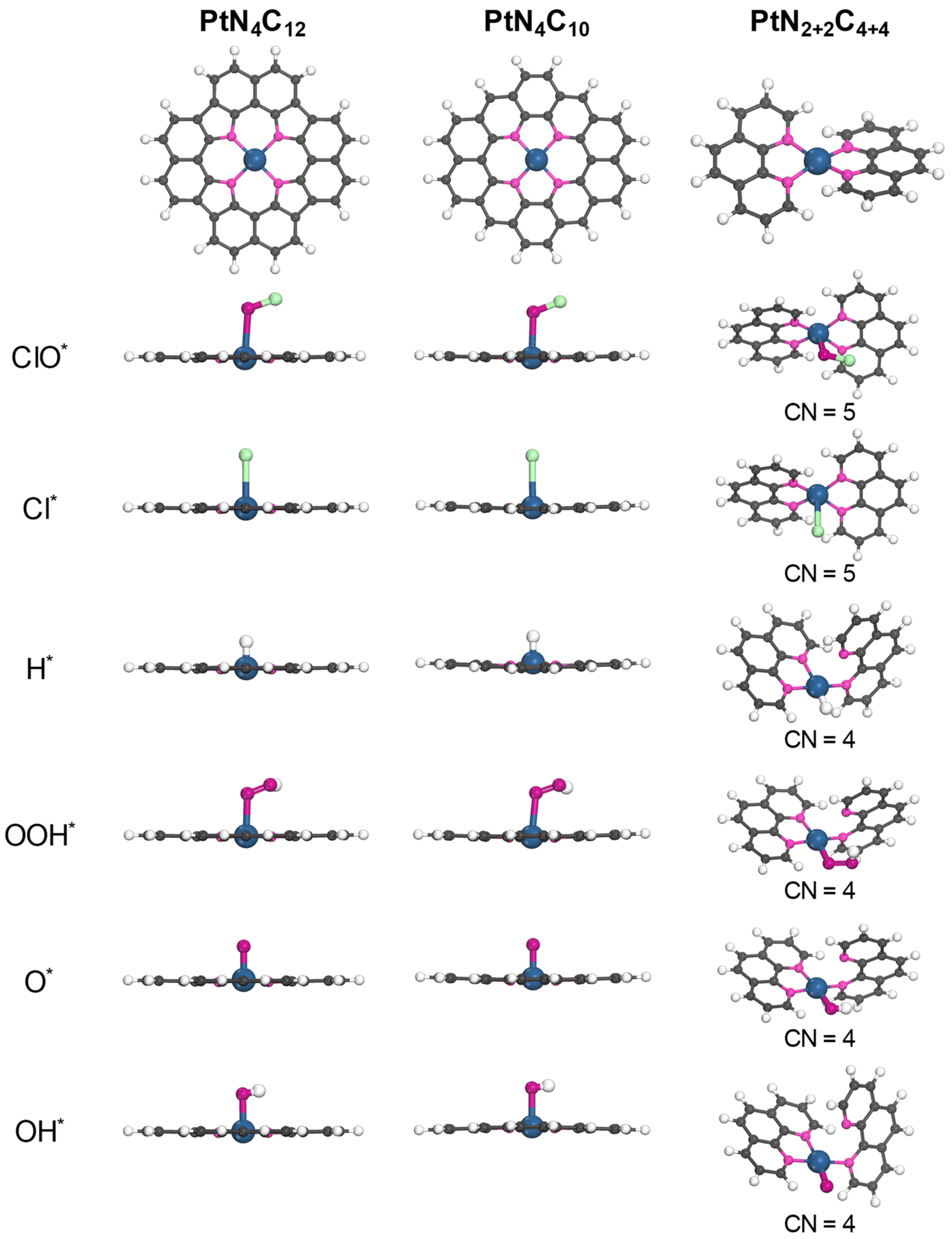

Supplementary Fig. 24 | Model systems for plausible Pt−N4 sites including PtN_4C_{12}, PtN_4C_{10}, and $PtN_{2+2}C_{4+4}$. Six plausible adsorbates (i.e., ClO^*, Cl^*, H^*, OOH^*, O^*, and OH^*) were considered. Coordination numbers (CNs) of Pt atoms in the $PtN_{2+2}C_{4+4}$ are labelled below. The white, black, pink, dark-blue, purple, and yellow-green coloured spheres represent the hydrogen, carbon, nitrogen, platinum, oxygen, and chlorine atoms, respectively.

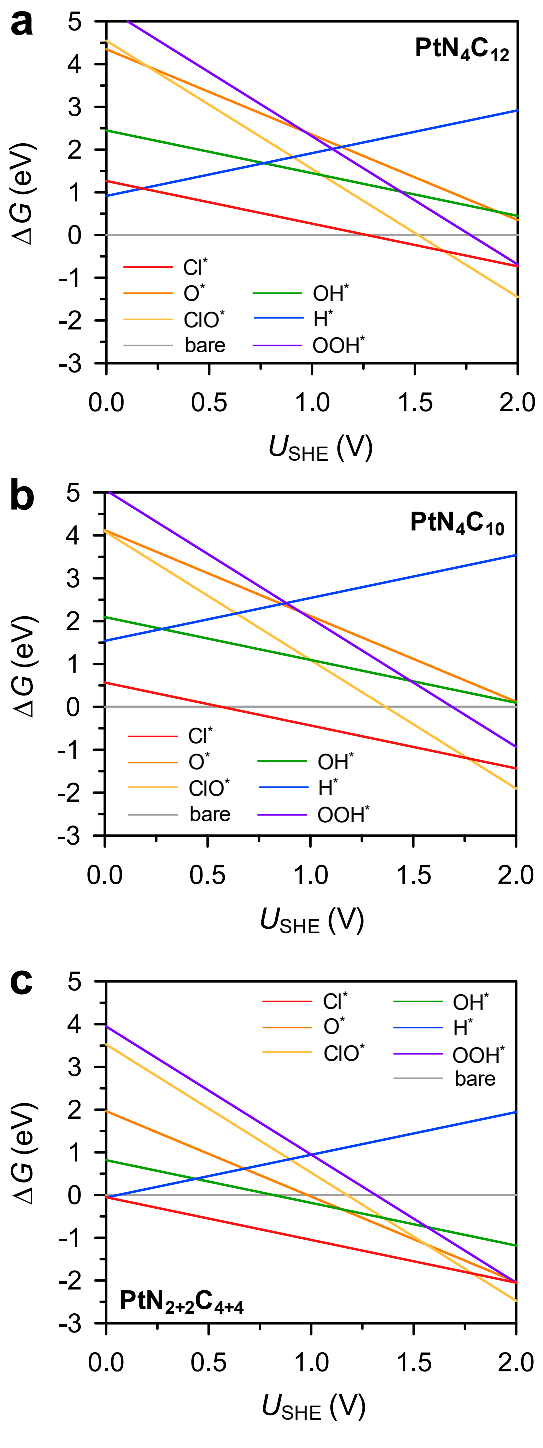

Supplementary Fig. 25 | The adsorption free energy for plausible adsorbates on three Pt−N4 sites. Seven plausible adsorbates (i.e., bare (*), Cl*, O*, ClO*, OH*, H*, and OOH*) on Pt−N4 sites were considered as a function of the theoretical standard hydrogen electrode potential (U_{SHE}) at pH = 0: **a** PtN_4C_{12}, **b** PtN_4C_{10}, and **c** $PtN_{2+2}C_{4+4}$.

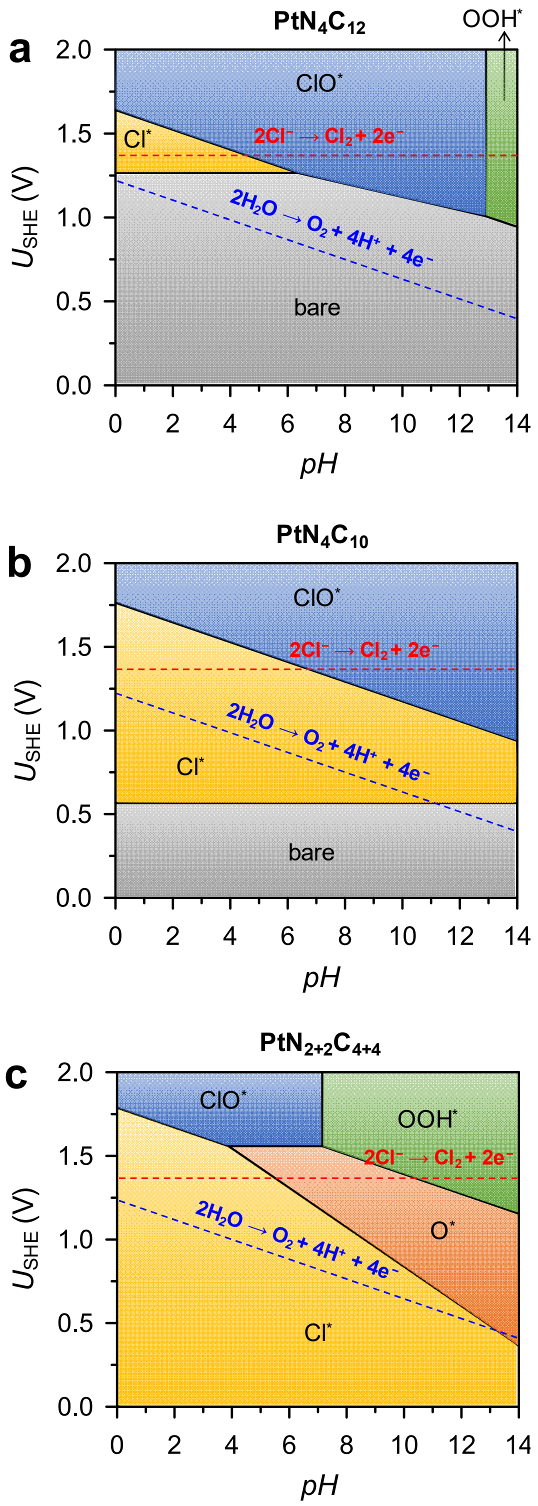

Supplementary Fig. 26 | Pourbaix diagram of theoretical standard hydrogen electrode potential (U_{SHE}) vs. pH for three Pt–N4 sites in equilibrium with H^+, Cl^- and H_2O at $T = 298$ K. **a** PtN_4C_{12}, **b** PtN_4C_{10}, and **c** $PtN_{2+2}C_{4+4}$. Red dashed line and blue dashed line represent the equilibrium potential for CER in the SHE scale (U_{eq} = 1.36 V) and OER (U_{eq} = 1.23 V –0.059 pH), respectively. Black solid lines represent the phase boundary where two adsorbate species exist in equilibrium.

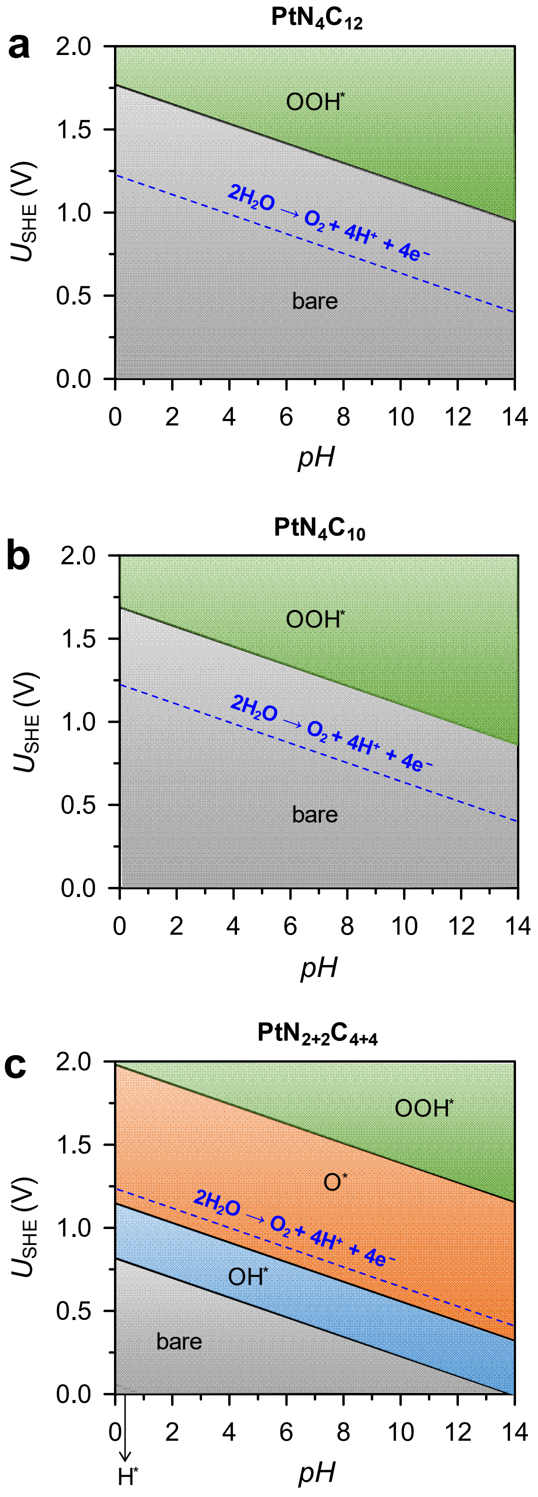

Supplementary Fig. 27 | Pourbaix diagram of theoretical standard hydrogen electrode potential (U_{SHE}) vs. pH for three Pt–N4 sites in equilibrium with H^+ and H_2O at $T = 298$ K. **a** PtN_4C_{12}, **b** PtN_4C_{10}, and **c** $PtN_{2+2}C_{4+4}$. Blue dashed line represents the equilibrium potential for OER in the SHE scale (i.e., $U_{eq} = 1.23$ V – 0.059 pH). Black solid lines represent the phase boundary where two adsorbate species exist in equilibrium.

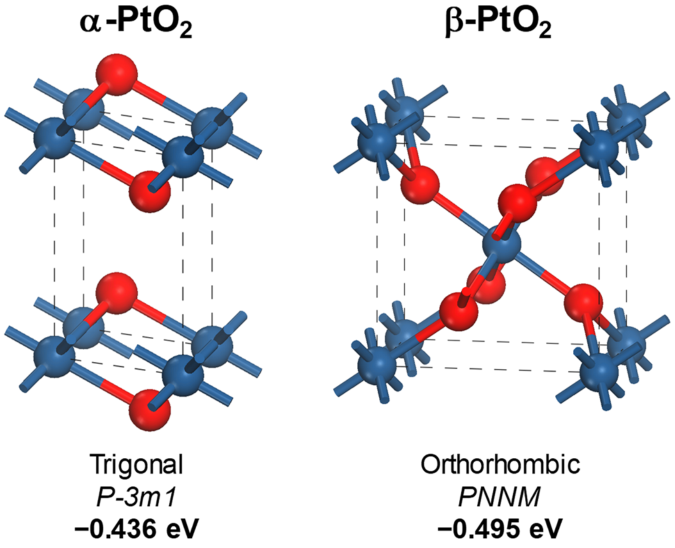

Supplementary Fig. 28 | Atomic structures of *α*- and *β*-phases of PtO_2. Crystalline system, space group, and Gibbs free energy of formation (ΔG_f) for each phase are given below the unit cells. The ΔG_f's are determined with respect to the bulk Pt and molecular oxygen (i.e., $\Delta G_f = G(PtO_2) - G(\text{Pt-bulk}) - G(O_{2(g)})$). Colour legends – Pt: dark-blue; O: red.

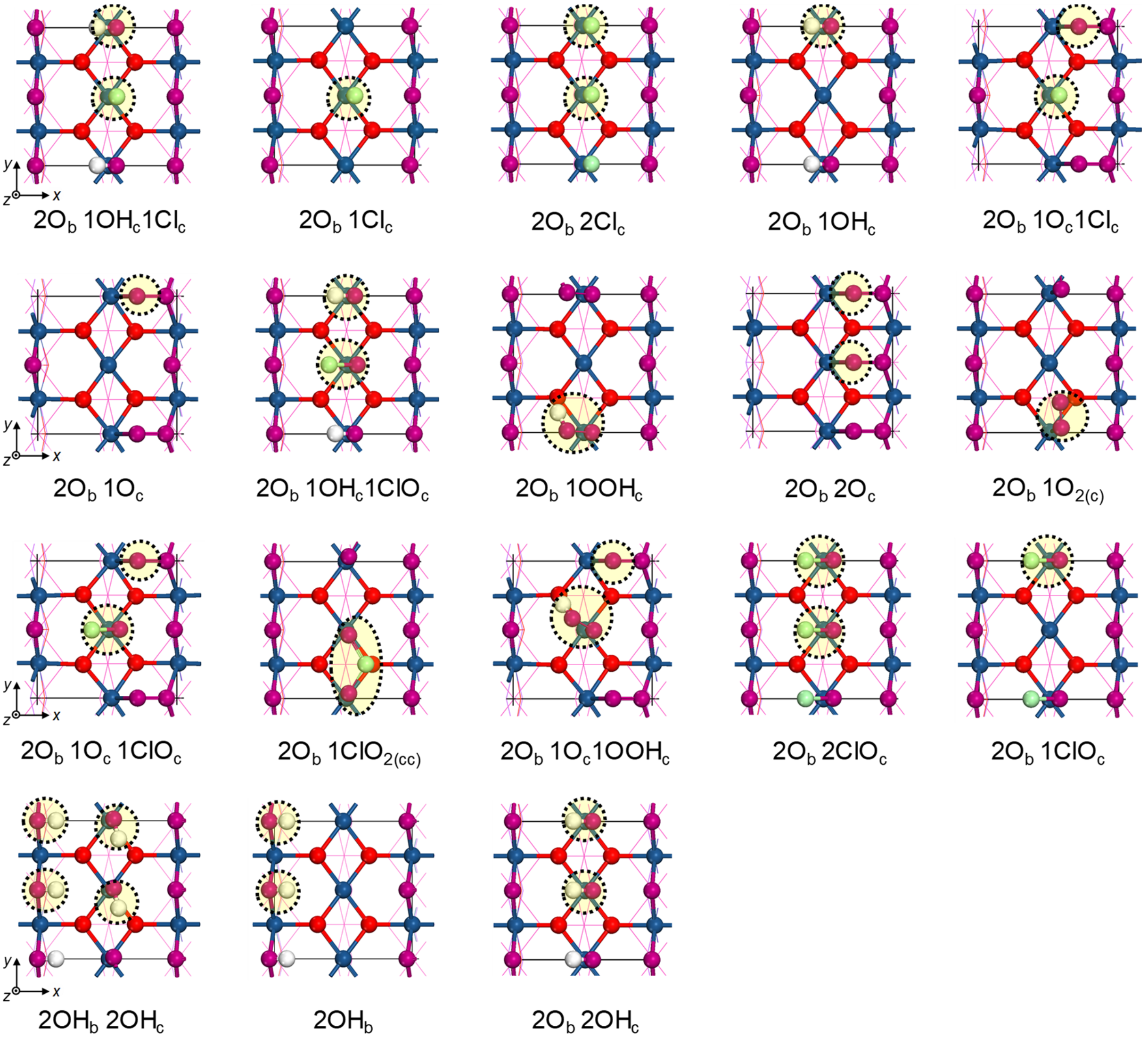

Supplementary Fig. 29 | Model systems for PtO_2 (110) surface including four plausible adsorption sites in top view. Among the four plausible adsorption sites, including two bridged oxygen and two coordinatively unsaturated (cus) sites, a total of 18 combinations of adsorbate species (i.e., O_b, OH_b at bridge sites as well as OH_c, Cl_c, O_c, OOH_c, $O_{2(c)}$, ClO_c at cus sites, respectively) were considered. Black dotted circles represent the adsorbate structures. Topmost layers are magnified with ball-and-stick style for visualisation. The dark-blue and red spheres represent the platinum and oxygen atoms at the surface, respectively. The white, yellow-green, and purple spheres represent the hydrogen, chlorine, and oxygen atoms for the adsorbates, respectively.

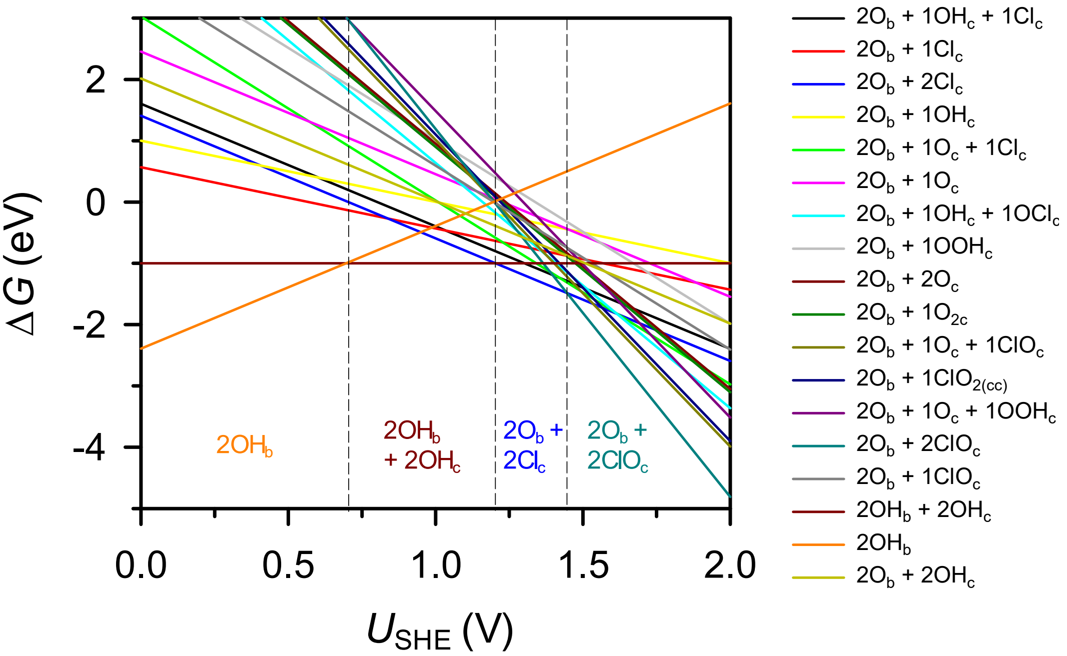

Supplementary Fig. 30 | Adsorption free energy (Δ*G*) of plausible adsorbates on PtO_2 (110) surface. A total of 18 plausible combinations of adsorbate species (i.e., O_b, OH_b at bridge sites as well as OH_c, Cl_c, O_c, OOH_c, $O_{2(c)}$, ClO_c at cus sites, respectively) were considered as a function of the theoretical standard hydrogen electrode potential (U_{SHE}) at pH = 0. Black dashed lines represent the phase boundary where two adsorbate species exist in equilibrium. The most stable adsorbate species at each area (divided by black dashed line) are labelled below.

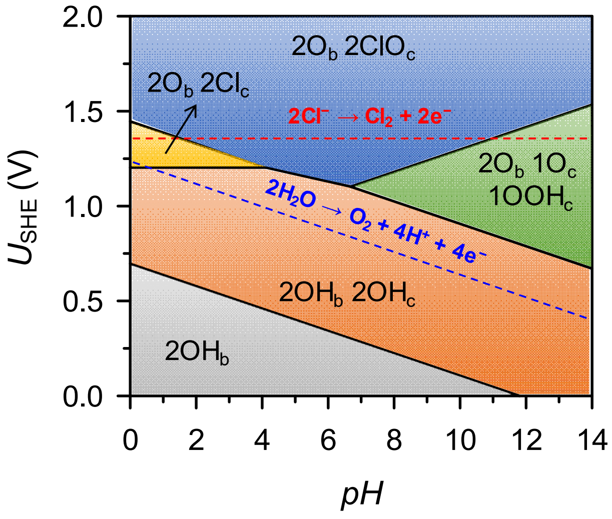

Supplementary Fig. 31 | Pourbaix diagram of theoretical standard hydrogen electrode potential (U_{SHE}) vs. pH for PtO_2 (110) surface. Red dashed line and blue dashed line represent the equilibrium potential for CER (U_{SHE} = 1.36 V) and OER, respectively. Black solid lines represent the phase boundary where two adsorbate species exist in equilibrium.

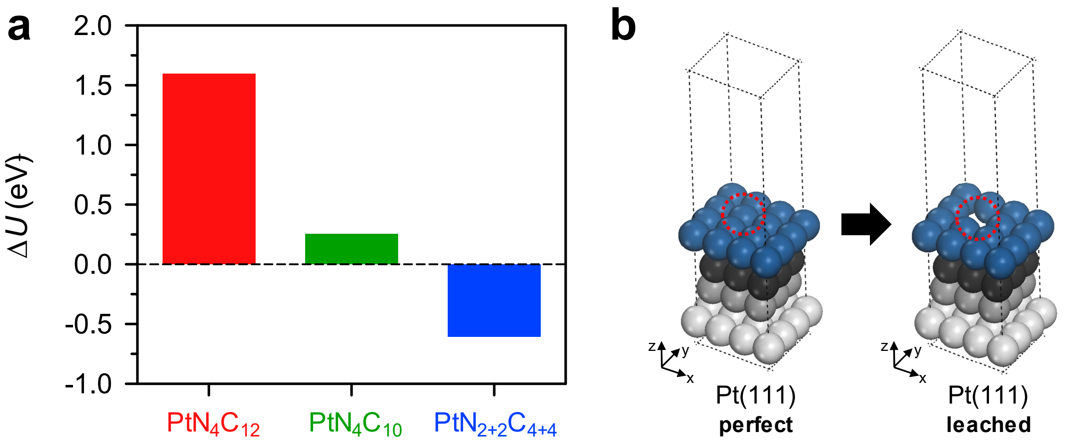

Supplementary Fig. 32 | **a** Electrode potential shift (ΔU), which was gauged with the difference in chemical potential of Pt atom in the Pt–N4 sites and that on the Pt(111) surface [i.e., $\mu_{\mathrm{Pt-N_4}} - \mu_{\mathrm{Pt(111)}}$]. The black dotted line represents the dissolution potential of Pt(111) surface. **b** Schematics for leaching of a Pt atom on the Pt(111) surface, used for $\mu_{\mathrm{Pt(111)}}$ calculation. Colour legends – Pt(top-most): dark-blue; Pt(second-most from top): black; Pt(third-most from top): grey; Pt(bottom-most): light-grey. The red dotted circle indicates the leaching site on the outermost layer.

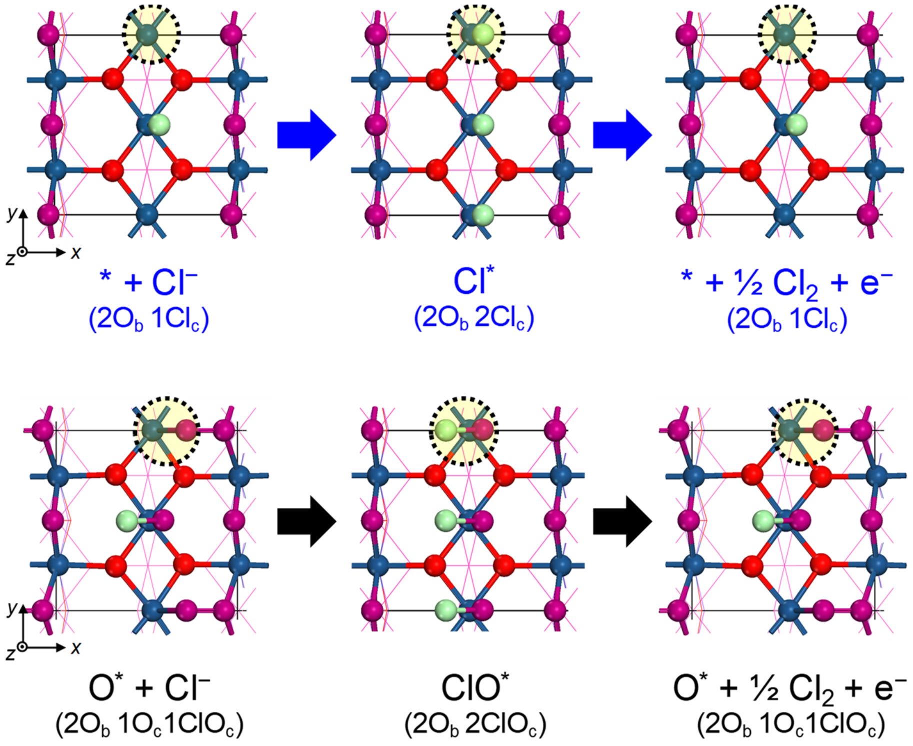

Supplementary Fig. 33 | Model systems for PtO_2 (110) surface for CER (top view). Two plausible intermediate structures including $2O_b2Cl_c$ and $2O_b2ClO_c$ were considered. Black dotted circles represent the reaction sites. Topmost layers are magnified with ball-and-stick style for visualisation. Dark-blue and red spheres represent the platinum and oxygen atoms at the surface, while the yellow-green and purple spheres represent the chlorine and oxygen atoms for the adsorbates, respectively.

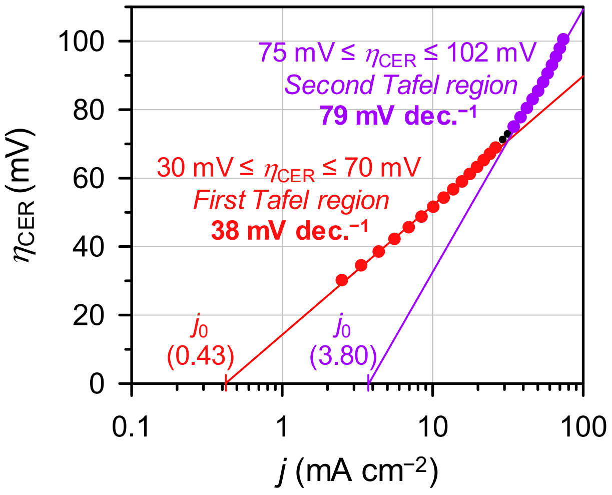

Supplementary Fig. 34 | Experimental Tafel plot exhibiting two linear Tafel regions with Tafel slopes of 38 and 79 mV dec^{-1}. The fitting ranges of overpotential for CER (η_{CER}) are indicated. The exchange current density j_0 is given by extrapolation.

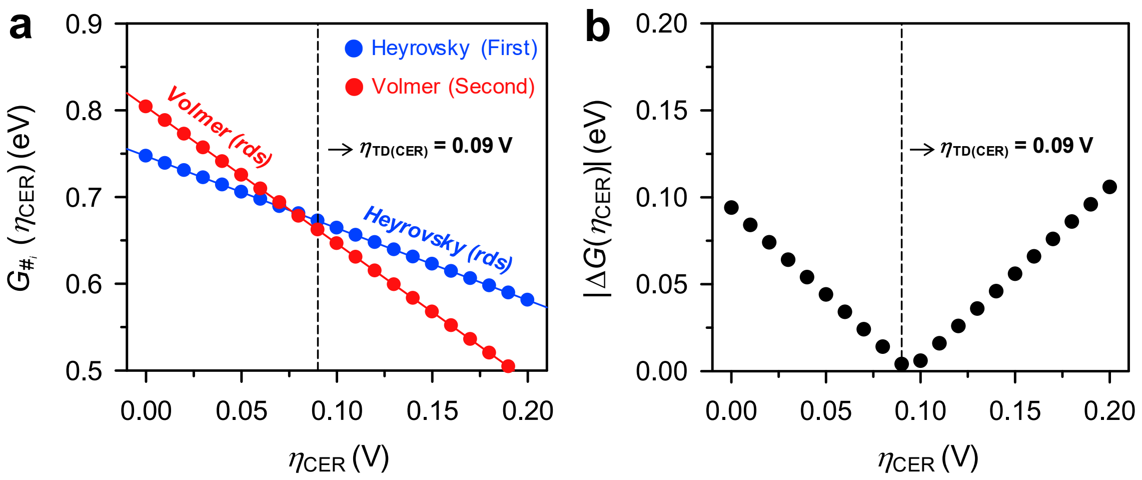

Supplementary Fig. 35 | Transition state free energy corresponding to the step $\#_i$ ($G_{\#_i}(\eta_{CER})$) as a function of applied overpotential η_{CER} in Pt_1/CNT. The first step ($\#_1$) corresponds to the Heyrovsky step, while the second step ($\#_2$) corresponds to the Volmer step. The black dashed line indicates the thermodynamic optimum of PtN_4C_{12} species, where the η_{CER} is equal to the thermodynamic overpotential for CER (i.e., $\eta_{CER} = \eta_{TD(CER)}$ (= 0.09 V)). **b** Absolute value of adsorption free energy for the reaction intermediate ($|\Delta G(\eta_{CER})|$) of PtN_4C_{12} species as a function of applied overpotential, η_{CER}. The black dashed line indicates the thermodynamic optimum ($|\Delta G(\eta_{CER})| = 0$), where the η_{CER} equals to the thermodynamic overpotential for CER (i.e., $\eta_{CER} = \eta_{TD(CER)}$ (= 0.09 V)).

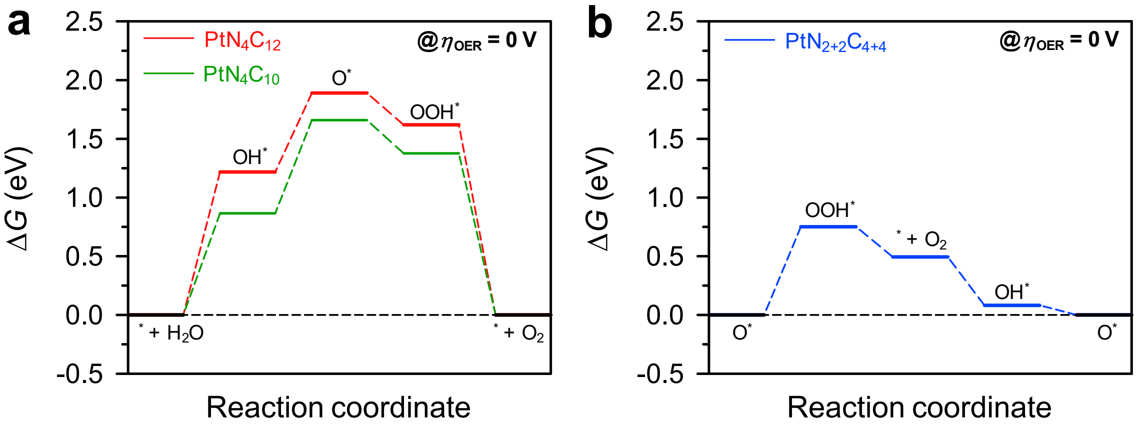

Supplementary Fig. 36 | Free energy diagrams for the OER on Pt−N4 sites at zero overpotential (η_{OER} = 0 V); **a** PtN_4C_{12}, PtN_4C_{10} and **b** $PtN_{2+2}C_{4+4}$. The active adsorbate structures for each model were employed as initial steps (i.e., bare structure (*) for PtN_4C_{12} and PtN_4C_{10}, and O^* for $PtN_{2+2}C_{4+4}$, respectively). The black dotted line represents the thermoneutral state (i.e., ΔG = 0).

Supplementary Table 1 | Elemental analysis results of CNT, Pt_1/CNT, and PtNP/CNT.

**Sample**	**C**	**N**	**O**	**H**	**Pt**
CNT	94.7<br>(95.62)	0.0<br>(0.0)	2.6<br>(1.97)	0.2<br>(2.41)	-<br>(-)
Pt_1/CNT	93.9<br>(94.86)	0.7<br>(0.61)	1.0<br>(0.76)	0.3<br>(3.61)	2.7<br>(0.17)
PtNP/CNT	93.3<br>(94.74)	0.0<br>(0.0)	1.9<br>(1.45)	0.3<br>(3.63)	2.9<br>(0.18)

C, **N**, **O**, and **H** were determined by EA and **Pt** was determined by ICP-OES. The contents of each element are presented in wt% with at% in parentheses.

Supplementary Table 2 | Summary of EXAFS fitting parameters of Pt_1/CNT catalyst, PtTPP precursor, PtNP/CNT catalyst, and Pt foil.

Sample	Shell	CN	*R* (Å)	σ^2 (10^{-3} Å^{-2})	ΔE_0 (eV)	*R* factor (%)
Pt_1/CNT	Pt−N	3.9 ± 0.7	2.00 ± 0.01	3.44 ± 1.49	12.51 ± 2.13	1.8
	Pt···C	4.0 ± 2.7	3.00 ± 0.03	5.03 ± 6.26		
PtTPP	Pt−N	4*	2.01 ± 0.01	1.09 ± 0.46	11.44 ± 1.80	0.9
	Pt···C	8*	3.04 ± 0.01	1.89 ± 0.99		
	∠Pt···C	16*	3.30 ± 0.04	1.28 ± 6.78		
PtNP/CNT	Pt−C	3.3 ± 0.9	2.04 ± 0.02	5.47 ± 2.48	4.25 ± 3.07	2.0
	Pt−Pt (1st)	7.7 ± 3.7	2.70 ± 0.02	17.33 ± 4.23		
Pt foil	Pt−Pt (1st)	12	2.77 ± 0.00	4.82 ± 0.10	8.28 ± 0.28	0.1
	Pt−Pt (2nd)	6	3.89 ± 0.01	4.79 ± 0.49		

Pt−N indicates a single scattering path of the first shell. Pt···C and ∠Pt···C indicate a single scattering path of the second shell and an obtuse triangle path of Pt···C, respectively (**Shell** column). The **CN** is the coordination number obtained from the amplitude reduction factor (S_0^2) of 0.85. * denotes the fixed constant value of **CN** obtained from the crystallographic data in a previous report[2]. ***R*** indicates bond distance. σ^2 indicates the Debye-Waller factor. ΔE_0 indicates the energy shift. ***R* factor** was obtained from the best fit for the respective catalysts.

Supplementary Table 3 | Comparison of CER activity and operation condition of Pt_1/CNT catalyst and those of previously reported catalysts in acidic media.

**Catalysts**	**Overpotential @10 mA cm^{-2} (mV)**	**Exchange current density (mA cm^{-2})**	**CER operation conditions**	**Precious metal contents**	**Ref.**
Pt_1/CNT (RRDE method)	50	0.43	0.1 M $HClO_4$ + 1.0 M NaCl (pH 0.9, 25 °C)	2.7 wt% Pt (0.17 at% Pt)	This work (Fig. 2a)
Pt_1/CNT (carbon paper)	70	0.44			This work (Supplementary Fig. 12a)
PtNP/CNT (RRDE method)	120	0.23	0.1 M $HClO_4$ + 1.0 M NaCl (pH 0.9, 25 °C)	2.9 wt% Pt (0.18 at% Pt)	This work (Fig. 2a)
Commercial DSA, Ru−Ti−Ir/Ti (Siontech, Korea)	105	0.20	0.1 M $HClO_4$ + 1.0 M NaCl (pH 0.9, 25 °C)	NA	This work (Supplementary Fig. 12a)
RuO_2 (110)	140*	4.5×10^{-3}	0.1 M $HClO_4$ + 1.0 M NaCl (pH 0.9, 25 °C)	NA	Supplementary Ref. [3]
Commercial DSA, Ru−Ti−Ir/Ti (Covestro, Germany)	90*	NA	3.0 M $NaNO_3$ + 1.0 M NaCl (pH 3.0, 25 °C)	NA	Supplementary Ref. [4]
Commercial DSA, $Ru_{0.3}Ti_{0.7}O_2$/Ti (Covestro, Germany)	180	NA	3.5 M NaCl (pH 3.0, 80 °C)	30 at% Ru	Supplementary Ref. [5]
Mesoporous Ru−Ir/TiO_2	140*	NA	4.0 M NaCl (pH 3.0, 25 °C)	7.5 wt% Ru 7.5 wt% Ir	Supplementary Ref. [6]
$RuTiO_x$/$SbSnO_x$	110	NA	5.0 M NaCl (pH 2.0, 25 °C)	NA	Supplementary Ref. [7]

* denotes the value of overpotential without considering *iR* compensation. **CER operation conditions** include Cl^- concentration, pH value, and temperature.

Supplementary Table 4 | Charge transfer resistances (R_{ct}) of Pt_1/CNT_*X* catalysts determined by the EIS fitting. The error indicates the standard deviation from the corresponding fitting results based on three independent measurements.

Catalysts	R_{ct} (Ω)
Pt_1/CNT_mix	445.9 ± 2.7
Pt_1/CNT_500	119.3 ± 0.9
Pt_1/CNT_600	21.3 ± 0.6
Pt_1/CNT_700	14.8 ± 0.4
Pt_1/CNT_800	13.3 ± 0.5

Supplementary Table 5 | Adsorption free energies (Δ*G*'s) of OH^*, O^*, and OOH^* on Pt−N_4 sites (i.e., PtN_4C_{12}, PtN_4C_{10}, and $PtN_{2+2}C_{4+4}$) and thermodynamic overpotentials for OER at zero overpotential ($\eta_{TD(OER)}$) at the overpotential (η_{OER}) of 0 and 0.13 V.

Sites	ΔG_{OH^*} (eV)	ΔG_{O^*} (eV)	ΔG_{OOH^*} (eV)	$\eta_{TD(OER)}$ (V)	$\eta_{TD(OER)}$ (V) (@η_{OER} = 0.13 V)
PtN_4C_{12}	2.45	4.35	5.31	1.22	1.09
PtN_4C_{10}	2.10	4.12	5.07	0.87	0.74
$PtN_{2+2}C_{4+4}$	0.82	1.97	3.95	0.75	0.62

Supplementary Note 1 | Calculation details and model systems (PtN_4C_{12}, PtN_4C_{10}, $PtN_{2+2}C_{4+4}$, and PtO_2 (110)).

Spin-polarised density functional theory (DFT) calculations were performed using the $DMol^3$ program[8,9]. The exchange-correlation energy was described by the generalised gradient approximation with the Perdew-Burke-Ernzerhof (GGA-PBE) functional[10]. The semi-empirical Tkatchenko-Scheffler (TS) approach[11] was applied to correct the van der Waals interactions. DFT semi-core pseudopotentials[12] were used for the core treatment. The double numerical polarisation (DNP) 4.4 level was employed as the basis set with an orbital cut-off of 4.5 Å. The implicit water environment was applied by the conductor-like screening model (COSMO)[13] using the dielectric constant of 78.54. For geometry optimisation, the convergence criteria were set to 1.0×10^{-5} for energy, 0.002 Ha $Å^{-1}$ for maximum force, and 0.005 Å for maximum displacement. For PtO_2 (110) surface, the Brillouin zone was sampled by the Monkhorst-Pack scheme[14] using 2×2×1 *k*-points where the dipole slab correction was applied. The self-consistent field tolerance for single-point energy calculation was set to 1.0×10^{-6} Ha with the thermal smearing parameter of 0.005 Ha.

Three molecular species (i.e., PtN_4C_{12}, PtN_4C_{10}, and $PtN_{2+2}C_{4+4}$) were chosen as the possible structural configurations of the Pt−N_4 sites (**Fig. 4a**). For the PtO_2 (110) surface model, a distorted rutile phase (referred to as β-PtO_2), which are predicted to be the most stable[15], was used for our study. First, the unit cell of β-PtO_2 was fully relaxed by DFT calculations (i.e., a = 4.58 Å, b = 4.61 Å, and c = 3.20 Å), where the lattice parameters were well matched with the experimental values[16]. Subsequently, a 2×1 supercell of PtO_2 (110) surface slab was modelled with nine atomic layers (3 O–Pt–O repeat units), where the bottom two layers were fixed to represent the bulk region (i.e., 6.41 Å × 6.49 Å × 27.1 Å). On top of the surface, four possible adsorption sites were present, including two coordinatively unsaturated (cus) sites and two bridging O sites. Following the previous studies for CER[17], we considered the possible adsorbates (i.e., ClO^*, Cl^*, H^*, OOH^*, O^*, and OH^*) and their relevant combinations for Pt−N_4 sites and PtO_2 (110) surface (**Supplementary Figs. 24 and 29**).

Supplementary Note 2 | Construction of Pourbaix diagrams of model systems.

The Pourbaix diagram represents the thermodynamically stable surface structures in electrochemical systems as a function of pH and electrode potential (*U*). Note that the detailed description about the Pourbaix diagram is reported elsewhere[18–20]. In this study, we constructed the Pourbaix diagrams for Pt–N_4 sites and PtO_2 (110) surface by calculating the adsorption free energies for all plausible adsorbates (i.e., H^*, O^*, OH^*, OOH^*, Cl^*, ClO^*, and $ClO_2{}^*$, see **Supplementary Figs. 24 and 29**). Note that * denotes the adsorbed states on the surface. A generalised description for the adsorbates (denoted as $O_mH_nCl_k$) on the site (denoted as *S*) can be written as

$$S^* + m\mathrm{H_2O} + k(\mathrm{Cl^-} - e^-) \rightleftharpoons S\text{-}\mathrm{O}_m\mathrm{H}_n\mathrm{Cl}_k{}^* + (2m - n)(\mathrm{H^+} + e^-) \tag{1}$$

By rewriting equation (1), each reaction can be represented as follows.

$$S^* \rightleftharpoons S\text{-}\mathrm{H}^* - (\mathrm{H^+} + \mathrm{e^-}) \quad (m = 0,\ n = 1,\ k = 0) \tag{2}$$

$$S^* + \mathrm{H_2O} \rightleftharpoons S\text{-}\mathrm{O}^* + 2(\mathrm{H^+} + \mathrm{e^-}) \quad (m = 1,\ n = 0,\ k = 0) \tag{3}$$

$$S^* + \mathrm{H_2O} \rightleftharpoons S\text{-}\mathrm{OH}^* + (\mathrm{H^+} + \mathrm{e^-}) \quad (m = 1,\ n = 1,\ k = 0) \tag{4}$$

$$S^* + 2\mathrm{H_2O} \rightleftharpoons S\text{-}\mathrm{OOH}^* + 3(\mathrm{H^+} + \mathrm{e^-}) \quad (m = 2,\ n = 1,\ k = 0) \tag{5}$$

$$S^* + (\mathrm{Cl^-} - \mathrm{e^-}) \rightleftharpoons S\text{-}\mathrm{Cl}^* \quad (m = 0,\ n = 0,\ k = 1) \tag{6}$$

$$S^* + \mathrm{H_2O} + (\mathrm{Cl^-} - \mathrm{e^-}) \rightleftharpoons S\text{-}\mathrm{ClO}^* + 2(\mathrm{H^+} + \mathrm{e^-}) \quad (m = 1,\ n = 0,\ k = 1) \tag{7}$$

$$S^* + 2\mathrm{H_2O} + (\mathrm{Cl^-} - \mathrm{e^-}) \rightleftharpoons S\text{-}\mathrm{ClO_2}{}^* + 4(\mathrm{H^+} + \mathrm{e^-}) \quad (m = 2,\ n = 0,\ k = 1) \tag{8}$$

where S^* denotes the bare site without adsorbates. Then, the adsorption free energy (ΔG) for $O_mH_nCl_k$ species can be defined as follows.

$$\Delta G = G_{S\text{-}\mathrm{O}_m\mathrm{H}_n\mathrm{Cl}_k{}^*} + (2m - n)(G_{\mathrm{H^+}} + G_{e^-}) - k(G_{\mathrm{Cl^-}} - G_{\mathrm{e^-}}) - G_{S^*} - mG_{\mathrm{H_2O}} \tag{9}$$

where *m*, *n*, and *k* denote the number of oxygen, hydrogen, and chlorine atoms, respectively. From DFT calculation, the ΔG for each adsorbate species can be calculated as follows.

$$\Delta G = \Delta E + \Delta ZPE - T\Delta S \tag{10}$$

where ΔE is the binding energy for each adsorbate, ΔZPE is the change in zero-point vibrational enthalpy, and $-T\Delta S$ is the entropic correction at room temperature.
By rewriting equation (9), the ΔG for each species can be represented as follows.

$$\Delta G_{\mathrm{H}^*} = G_{S\text{-}\mathrm{H}^*} - (G_{\mathrm{H^+}} + G_{\mathrm{e^-}}) - G_{S^*} \quad (m = 0,\ n = 1,\ k = 0) \tag{11}$$

$$\Delta \mathrm{G}_{\mathrm{O}^*} = G_{S\text{-}\mathrm{O}^*} + 2(G_{\mathrm{H^+}} + G_{\mathrm{e^-}}) - G_{S^*} - G_{\mathrm{H_2O}} \quad (m = 1,\ n = 0,\ k = 0) \tag{12}$$

$$\Delta G_{\mathrm{OH}^*} = G_{S\text{-}\mathrm{OH}^*} + (G_{\mathrm{H^+}} + G_{\mathrm{e^-}}) - G_{S^*} - G_{\mathrm{H_2O}} \quad (m = 1,\ n = 1,\ k = 0) \tag{13}$$

$$\Delta G_{\mathrm{OOH}^*} = G_{S\text{-}\mathrm{OOH}^*} + 3(G_{\mathrm{H^+}} + G_{\mathrm{e^-}}) - G_{S^*} - 2G_{\mathrm{H_2O}} \quad (m = 2,\ n = 1,\ k = 0) \tag{14}$$

$$\Delta G_{\mathrm{Cl}^*} = G_{S\text{-}\mathrm{Cl}^*} - (G_{\mathrm{Cl}^-} - G_{\mathrm{e}^-}) - G_{S^*} \qquad (m = 0,\ n = 0,\ k = 1) \tag{15}$$

$$\Delta G_{\mathrm{ClO}^*} = G_{S\text{-}\mathrm{ClO}^*} + 2(G_{\mathrm{H}^+} + G_{\mathrm{e}^-}) - (G_{\mathrm{Cl}^-} - G_{\mathrm{e}^-}) - G_{S^*} - G_{\mathrm{H_2O}} \qquad (m = 1,\ n = 0,\ k = 1) \tag{16}$$

$$\Delta G_{\mathrm{ClO_2}^*} = G_{S\text{-}\mathrm{ClO_2}^*} + 4(G_{\mathrm{H}^+} + G_{\mathrm{e}^-}) - (G_{\mathrm{Cl}^-} - G_{\mathrm{e}^-}) - G_{S^*} - 2G_{\mathrm{H_2O}} \qquad (m = 2,\ n = 0,\ k = 1) \tag{17}$$

Herein, $G_{\mathrm{H}^+} + G_{\mathrm{e}^-}$ and $G_{\mathrm{Cl}^-} - G_{\mathrm{e}^-}$ is defined as a function of U_{SHE}, pH, and $ln\, a_{\mathrm{Cl}^-}$.

$$G_{\mathrm{H}^+} + G_{\mathrm{e}^-} = \frac{1}{2} G_{\mathrm{H_2}} - U_{\mathrm{SHE}} - \ln 10 \cdot k_{\mathrm{B}} T \cdot \mathrm{pH} \tag{18}$$

$$G_{\mathrm{Cl}^-} - G_{\mathrm{e}^-} = \frac{1}{2} G_{\mathrm{Cl_2}} + U_{\mathrm{SHE}} - 1.36 + \ln a_{\mathrm{Cl}^-} \cdot k_{\mathrm{B}} T \tag{19}$$

where U_{SHE}, k_{B}, and T denote the electrode potential (vs. theoretical standard hydrogen electrode, SHE), Boltzmann constant, and temperature, respectively. Considering that the Cl^- concentration is nearly constant under reaction condition by using $NaClO_4$ as a buffer solution, $\ln a_{\mathrm{Cl}^-}$ was assumed to be negligible for our calculation[17].
At finite U_{SHE} and pH, the ΔG can be expressed as

$$\begin{aligned} \Delta G(U, \mathrm{pH}) = G_{S\text{-}\mathrm{O}_m\mathrm{H}_n\mathrm{Cl}_k{}^*} &+ (2m - n)\left(\frac{1}{2} G_{\mathrm{H_2}} - U_{\mathrm{SHE}} - \ln 10 \cdot k_{\mathrm{B}} T \cdot \mathrm{pH}\right) \\ &- k\left(\frac{1}{2} G_{\mathrm{Cl_2}} + U_{\mathrm{SHE}} - 1.36\right) - G_{S^*} - m G_{\mathrm{H_2O}} \end{aligned} \tag{20}$$

By rewriting equation (20), the ΔG for each species as a function of U_{SHE} and pH can be defined as follows at standard conditions ($T = 298$ K).

$$\begin{aligned} \Delta G_{\mathrm{H}^*}(U, \mathrm{pH}) &= G_{S\text{-}\mathrm{H}^*} - \left(\frac{1}{2} G_{\mathrm{H_2}} - U_{\mathrm{SHE}} - \ln 10 \cdot k_{\mathrm{B}} T \cdot \mathrm{pH}\right) - G_{S^*} \\ &= \Delta G_{\mathrm{H}^*} + U_{\mathrm{SHE}} + 0.059 \cdot \mathrm{pH} \end{aligned} \tag{21}$$

$$\begin{aligned} \Delta G_{\mathrm{O}^*}(U, \mathrm{pH}) &= G_{S\text{-}\mathrm{O}^*} + 2\left(\frac{1}{2} G_{\mathrm{H_2}} - U_{\mathrm{SHE}} - \ln 10 \cdot k_{\mathrm{B}} T \cdot \mathrm{pH}\right) - G_{S^*} - G_{\mathrm{H_2O}} \\ &= \Delta G_{\mathrm{O}^*} - 2U_{\mathrm{SHE}} - 0.118 \cdot \mathrm{pH} \end{aligned} \tag{22}$$

$$\begin{aligned} \Delta G_{\mathrm{OH}^*}(U, \mathrm{pH}) &= G_{S\text{-}\mathrm{OH}^*} + \left(\frac{1}{2} G_{\mathrm{H_2}} - U_{\mathrm{SHE}} - \ln 10 \cdot k_{\mathrm{B}} T \cdot \mathrm{pH}\right) - G_{S^*} - G_{\mathrm{H_2O}} \\ &= \Delta G_{\mathrm{OH}^*} - U_{\mathrm{SHE}} - 0.059 \cdot \mathrm{pH} \end{aligned} \tag{23}$$

$$\begin{aligned} \Delta G_{\mathrm{OOH}^*}(U, \mathrm{pH}) &= G_{S\text{-}\mathrm{OOH}^*} + 3\left(\frac{1}{2} G_{\mathrm{H_2}} - U_{\mathrm{SHE}} - \ln 10 \cdot k_{\mathrm{B}} T \cdot \mathrm{pH}\right) - G_{S^*} - 2G_{\mathrm{H_2O}} \\ &= \Delta G_{\mathrm{OOH}^*} - 3U_{\mathrm{SHE}} - 0.177 \cdot \mathrm{pH} \end{aligned} \tag{24}$$

$$\begin{aligned} \Delta G_{\mathrm{Cl}^*}(U, \mathrm{pH}) &= G_{S\text{-}\mathrm{Cl}^*} - \left(\frac{1}{2} G_{\mathrm{Cl_2}} + U_{\mathrm{SHE}} - 1.36\right) - G_{S^*} \\ &= \Delta G_{\mathrm{Cl}^*} - U_{\mathrm{SHE}} + 1.36 \end{aligned} \tag{25}$$

$$\begin{aligned} \Delta G_{\mathrm{ClO}^*}(U, \mathrm{pH}) &= G_{S\text{-}\mathrm{ClO}^*} + 2\left(\frac{1}{2} G_{\mathrm{H_2}} - U_{\mathrm{SHE}} - \ln 10 \cdot k_{\mathrm{B}} T \cdot \mathrm{pH}\right) \\ &\quad - \left(\frac{1}{2} G_{\mathrm{Cl_2}} + U_{\mathrm{SHE}} - 1.36\right) - G_{S^*} - G_{\mathrm{H_2O}} \\ &= \Delta G_{\mathrm{ClO}^*} - 3U_{\mathrm{SHE}} + 1.36 - 0.118 \cdot \mathrm{pH} \end{aligned} \tag{26}$$

$$\Delta G_{\mathrm{ClO_2}^*}(U, \mathrm{pH}) = G_{S\text{-}\mathrm{ClO_2}^*} + 4\left(\frac{1}{2} G_{\mathrm{H_2}} - U_{\mathrm{SHE}} - \ln 10 \cdot k_{\mathrm{B}} T \cdot \mathrm{pH}\right)$$

$$-\left(\frac{1}{2}G_{\mathrm{Cl_2}} + U_{\mathrm{SHE}} - 1.36\right) - G_{S^*} - 2G_{\mathrm{H_2O}}$$
$$= \Delta G_{\mathrm{ClO_2}^*} - 5U_{\mathrm{SHE}} + 1.36 - 0.236 \cdot \mathrm{pH} \qquad (27)$$

In this study, ΔG's for all species as a function of U_{SHE} were initially found to determine the phase boundaries at pH = 0 (**Supplementary Figs. 25 and 30**). Subsequently, by applying the effect of pH to the ΔG's, the Pourbaix diagrams for the Pt−N_4 sites and PtO_2 (110) surface were finally constructed (**Supplementary Figs. 26, 27, and 31**).

Supplementary Note 3 | Free energy diagram of model systems for CER and OER.

To theoretically investigate the CER activity, we calculated the free energy diagrams for the CER in the Pt–N_4 sites and PtO_2 (110) surface (**Fig. 4c**). To accomplish this, two possible reaction mechanisms including different intermediates (i.e., Cl^* or ClO^*) were considered.

(I) Pathway mediated by the Cl^* species

$$S^* + \mathrm{Cl}^-(aq) \rightleftharpoons S\text{-Cl}^* + e^- \tag{28}$$

$$S\text{-Cl}^* + \mathrm{Cl}^-(aq) \rightleftharpoons S^* + \mathrm{Cl}_2(g) + e^- \tag{29}$$

(II) Pathway mediated by the ClO^* species

$$S\text{-O}^* + \mathrm{Cl}^-(aq) \rightleftharpoons S\text{-ClO}^* + e^- \tag{30}$$

$$S\text{-ClO}^* + \mathrm{Cl}^-(aq) \rightleftharpoons S\text{-O}^* + \mathrm{Cl}_2(g) + e^- \tag{31}$$

where (aq) and (g) represent the aqueous and gaseous phases, respectively.
The ΔE's for each reaction intermediate were calculated relative to the Cl_2 gas molecule as follows.

$$\Delta E_{\mathrm{Cl}^*} = E_{S\text{-Cl}^*} - E_{S^*} - 0.5E_{\mathrm{Cl}_2} \tag{32}$$

$$\Delta E_{\mathrm{ClO}^*} = E_{S\text{-ClO}^*} - E_{S^*} - 0.5E_{\mathrm{Cl}_2} - (E_{\mathrm{H_2O}} - E_{\mathrm{H_2}}) \tag{33}$$

Using equations (10), (12), (15), and (16), ΔG_{Cl^*}, ΔG_{ClO^*}, and ΔG_{O^*} can be calculated. Then, the thermodynamic overpotential for CER at zero overpotential ($\eta_{TD(CER)}$) can be defined as follows.

(I) For * and Cl^* species

$$\eta_{\mathrm{TD(CER)}} = \frac{|\Delta G_{\mathrm{Cl}^*}|}{e} \tag{34}$$

(II) For * and ClO^* species

$$\eta_{\mathrm{TD(CER)}} = \frac{|\Delta G_{\mathrm{ClO}^*} - \Delta G_{\mathrm{O}^*}|}{e} \tag{35}$$

To investigate the thermodynamic overpotential for OER at the zero overpotential ($\eta_{TD(OER)}$) of Pt–N_4 sites, we assumed the conventional four-electron pathway for the OER as follows.

$$S^* + \mathrm{H_2O}(l) \rightleftharpoons S\text{-OH}^* + (\mathrm{H}^+ + \mathrm{e}^-) \tag{36}$$

$$S\text{-OH}^* \rightleftharpoons S\text{-O}^* + (\mathrm{H}^+ + \mathrm{e}^-) \tag{37}$$

$$S\text{-O}^* + \mathrm{H_2O}(l) \rightleftharpoons S\text{-OOH}^* + (\mathrm{H}^+ + \mathrm{e}^-) \tag{38}$$

$$S\text{-OOH}^* \rightleftharpoons S^* + \mathrm{O_2}(g) + (\mathrm{H}^+ + \mathrm{e}^-) \tag{39}$$

where (l) indicates the liquid phase. The ΔE's for each reaction intermediate were calculated relative to H_2O and H_2 molecules as follows.

$$\Delta E_{\mathrm{OH}^*} = E_{S\text{-OH}^*} - E_{S^*} - (E_{\mathrm{H_2O}} - 0.5E_{\mathrm{H_2}}) \quad (40)$$

$$\Delta E_{\mathrm{O}^*} = E_{S\text{-O}^*} - E_{S^*} - (E_{\mathrm{H_2O}} - E_{\mathrm{H_2}}) \quad (41)$$

$$\Delta E_{\mathrm{OOH}^*} = E_{S\text{-OOH}^*} - E_{S^*} - (2E_{\mathrm{H_2O}} - 1.5E_{\mathrm{H_2}}) \quad (42)$$

where $E_{\mathrm{S\text{-}OH}^*}$, $E_{\mathrm{S\text{-}O}^*}$, and $E_{\mathrm{S\text{-}OOH}^*}$ are the total energies of adsorbed state of the Pt–N_4 sites (i.e., OH^*, O^*, and OOH^*, respectively); E_{S^*} is the total energy of bare state of the Pt–N_4 sites; $E_{\mathrm{H_2O}}$ and $E_{\mathrm{H_2}}$ are the total energies of isolated water molecules and hydrogen gas, respectively. The reaction free energy of equations (43–46) (ΔG_1, ΔG_2, ΔG_3, ΔG_4) for OER can be calculated as follows.

$$\Delta G_1 = \Delta G_{\mathrm{OH}^*} \quad (43)$$

$$\Delta G_2 = \Delta G_{\mathrm{O}^*} - \Delta G_{\mathrm{OH}^*} \quad (44)$$

$$\Delta G_3 = \Delta G_{\mathrm{OOH}^*} - \Delta G_{\mathrm{O}^*} \quad (45)$$

$$\Delta G_4 = 4.92 - \Delta G_{\mathrm{OOH}^*} \quad (46)$$

Finally, $\eta_{\mathrm{TD(OER)}}$ can be defined by

$$\eta_{\mathrm{TD(OER)}} = \frac{max[\Delta G_1, \Delta G_2, \Delta G_3, \Delta G_4]}{e} - U_{\mathrm{eq}}\ [\mathrm{V}] \quad (47)$$

where U_{eq} indicates the equilibrium potential for OER (i.e., 1.23 V vs. SHE).

Supplementary Note 4 | Stability against Pt dissolution for Pt−N₄ sites.

The extent of dissolution of Pt from Pt–N_4 sites relative to that from Pt(111) surface (**Supplementary Fig. 32**) could be gauged with the electrode potential shift (ΔU) for reaction $Pt \rightarrow Pt^{2+} + 2e^{-}$, by following the previous reports[21,22]. The ΔU is defined by

$$\Delta U = \frac{-[\mu_{\text{Pt-N}_4} - \mu_{\text{Pt(111)}}]}{2e} \quad (48)$$

where $\mu_{\text{Pt-N}_4}$ and $\mu_{\text{Pt(111)}}$ are the chemical potentials of Pt atoms on the Pt–N_4 sites and Pt(111) surface, respectively. Note that the dissolutions of Pt atoms are assumed to only occur on the outermost layer of the surface. Assuming the reference state as Pt bulk state, the $\mu_{\text{Pt-N}_4}$ and $\mu_{\text{Pt(111)}}$ were calculated as follows.

$$\mu_{\text{Pt-N}_4} = E_{\text{Pt-N}_4} - E_{\text{Pt-free}} - E_{\text{Pt-bulk}} \quad (49)$$

$$\mu_{\text{Pt(111)}} = E_{\text{Pt(111)}} - E_{\text{Pt-leached}} - E_{\text{Pt-bulk}} \quad (50)$$

where $E_{\text{Pt-N}_4}$, $E_{\text{Pt-free}}$, $E_{\text{Pt-bulk}}$, $E_{\text{Pt(111)}}$, and $E_{\text{Pt-leached}}$ represent the DFT-optimised energies of Pt–N_4 sites, Pt–N_4 sites where the Pt atom is not included, a Pt atom in the bulk unit cell, perfect Pt(111) surface, and Pt(111) surface where a single Pt atom on the outermost surface is leached, respectively.

Supplementary Note 5 | Full free energy diagram for CER over Pt_1/CNT.

By combining the experimental data for the kinetics and theoretical data for thermodynamics, a full free energy diagram along the reaction coordinate of CER over Pt_1/CNT was constructed. Details regarding the definition and derivation of this approach are fully given in the earlier works by Exner and co-workers[23,24]. Within the Butler-Volmer formalism, the Tafel slope, b, is defined by the following equation.

$$b = \frac{k_B T \cdot \ln(10)}{e \cdot (\gamma + r_{rds}\alpha_k)} \tag{51}$$

where k_B is the Boltzmann's constant, T is the temperature, e is the elementary charge, γ is the integer number of transferred electrons before the rate-determining step (rds), r_{rds} is 0 for chemical (i.e., no charge transfer) and 1 for electrochemical step, and α_k is the transfer coefficient of the considered reaction step k. With increased overpotential for the CER (η_{CER}), the experimental Tafel plot revealed two linear Tafel regions with b of 38 mV dec.$^{-1}$ (30 mV $\leq \eta_{CER} \leq$ 70 mV) and 79 mV dec.$^{-1}$ (75 mV $\leq \eta_{CER} \leq$ 102 mV) (**Supplementary Fig. 34**). At room temperature, the respective γ, r_{rds}, and α_k for each Tafel region were determined as $\gamma = 0$, $r_{rds} = 1$, $\alpha_1 = 0.83$ (for first region, $k = 1$) and $\gamma = 1$, $r_{rds} = 1$, $\alpha_2 = 0.58$ (for second region, $k = 2$). The overall current density (j) can be expressed as a function of η_{CER}.

$$\log\big(j(\eta_{CER})\big) = \log\left(\frac{k_B T \cdot 2e\Gamma_{act}}{h}\right) - \frac{G^{\#}_{rds}}{k_B T \cdot \ln(10)} + \frac{\eta_{CER}}{b} = \log(j_0) + \frac{\eta_{CER}}{b} \tag{52}$$

where h is the Plank's constant, Γ_{act} is the number of active sites per area, $G^{\#}_{rds}$ is the free energy of transition state (TS) at the rds, and j_0 is the exchange current density. The value of Γ_{act} was obtained from the equation (53).

$$\Gamma_{act} = m \times N_A \tag{53}$$

where m is the molar number of Pt-catalyst loaded on the electrode (i.e., 14.00 nmol cm^{-2} for Pt_1/CNT) and N_A is the Avogadro's number (6.022×10^{23}). From the $\log(j_0)$, we can determine the $G^{\#}_{rds}$ as follows.

$$G^{\#}_{rds} = k_B T \cdot \ln(10)\left(\log\left(\frac{k_B T \cdot 2e\Gamma_{act}}{h}\right) - \log(j_0)\right) \tag{54}$$

The $G^{\#}_{rds}$'s were determined as 0.75 (for first TS) and 0.80 eV (for second TS) in the Pt_1/CNT. When the η_{CER} is applied ($\eta_{CER} > 0$), the free energies of each state are affected by the number of transferred electrons, z (i.e., $z = 0$, 1, and 2 for initial state (IS), intermediate state (IM), and final state (FS)) and α_k (i.e., $\alpha_1 = 0.83$ for first TS, and $(1+\alpha_2) = 1.58$ for second TS, respectively). Resultingly, with increasing η_{CER}, the free energies for the first TS, IM, second TS, and FS along the reaction coordinate of CER over Pt_1/CNT were lowered by $0.83 \cdot e \cdot \eta_{CER}$, $1 \cdot e \cdot \eta_{CER}$, $1.58 \cdot e \cdot \eta_{CER}$, and $2 \cdot e \cdot \eta_{CER}$, respectively (**Fig. 4d**).

The TS free energies of step $\#_i$ ($G_{\#_i}(\eta_{CER})$) showed that with increasing η_{CER}, the rds was switched from the first TS (Heyrovsky step, $i = 1$) to the second TS (Volmer step, $i = 2$) due to

the larger decrease in free energies by η_{CER} in the Volmer step (indicated by the slope, $\mathrm{d}G_{\#_i}(\eta_{\mathrm{CER}})\ \mathrm{d}\eta_{\mathrm{CER}}^{-1}$, in the **Supplementary Fig. 35a**). Additionally, the absolute value of free energy for the IM ($|\Delta G(\eta_{\mathrm{CER}})|$) showed that it reached the thermoneutral state at the point where η_{CER} is equal to the thermodynamic overpotential of CER (i.e., $\eta_{\mathrm{TD(CER)}}$ = 0.09 V for PtN_4C_{12} species, **Supplementary Fig. 35b**).

Supplementary References

1. Macquet, J. P., Millard, M. M. & Theophanides, T. X-Ray photoelectron spectroscopy of porphyrins. *J. Am. Chem. Soc.* **100**, 4741−4746 (1978).

2. Hazell, A. C. Structure of (5,10,15,20-tetraphenyl-21*H*,23*H*-porphinato)platinum(II), $C_{44}H_{28}N_4Pt$. *Acta Crystallogr. C* **40**, 751−753 (1984).

3. Sohrabnejad-Eskan, I. *et al.* Temperature-dependent kinetic studies of the chlorine evolution reaction over RuO_2(110) model electrodes. *ACS Catal.* **7**, 2403−2411 (2017).

4. Zeradjanin, A. R., Menzel, N., Schuhmann, W. & Strasser, P. On the faradaic selectivity and the role of surface inhomogeneity during the chlorine evolution reaction on ternary Ti−Ru−Ir mixed metal oxide electrocatalysts. *Phys. Chem. Chem. Phys.* **16,** 13741−13747 (2014).

5. Chen, R. *et al.* Microstructural impact of anodic coatings on the electrochemical chlorine evolution reaction. *Phys. Chem. Chem. Phys.* **14**, 7392−7399 (2012).

6. Menzel, N., Ortel, E., Mette, K., Kraehnert, R. & Strasser, P. Dimensionally stable Ru/Ir/TiO_2-anodes with tailored mesoporosity for efficient electrochemical chlorine evolution. *ACS Catal.* **3**, 1324−1333 (2013).

7. Moreno-Hernandez, I. A., Brunschwig, B. S. & Lewis, N. S. Crystalline nickel, cobalt, and manganese antimonates as electrocatalysts for the chlorine evolution reaction. *Energy Environ. Sci.* **12**, 1241−1248 (2019)

8. Delley, B. An all-electron numerical method for solving the local density functional for polyatomic molecules. *J. Chem. Phys.* **92**, 508–517 (1990).

9. Delley, B. From molecules to solids with the $DMol^3$ approach. *J. Chem. Phys.* **113**, 7756–7764 (2000).

10. Perdew, J. P., Burke, K. & Ernzerhof, M. Generalized gradient approximation made simple. *Phys. Rev. Lett.* **77**, 3865–3868 (1996).

11. Tkatchenko, A. & Scheffler, M. Accurate molecular Van Der Waals interactions from ground-state electron density and free-atom reference data. *Phys. Rev. Lett.* **102**, 073005 (2009).

12. Delley, B. Hardness conserving semilocal pseudopotentials. *Phys. Rev. B* **66**, 155125 (2002).

13. Klamt, A. & Schüürmann, G. COSMO: a new approach to dielectric screening in solvents with explicit expressions for the screening energy and its gradient. *J. Chem. Soc. Perkin Trans. 2* 799–805 (1993).

14. Monkhorst, H. J. & Pack, J. D. Special points for Brillouin-zone integrations. *Phys. Rev. B* **13**, 5188–5192 (1976).

15. Nomiyama, R. K., Piotrowski, M. J. & Da Silva, J. L. F. Bulk structures of PtO and PtO_2 from density functional calculations. *Phys. Rev. B* **84**, 100101 (2011).

16. Range, K.-J., Rau, F., Klement, U. & Heyns, A. M. β-PtO_2: high pressure synthesis of single crystals and structure refinement. *Mater. Res. Bull.* **22**, 1541−1547 (1987).

17. Exner, K. S., Anton, J., Jacob, T. & Over, H. Chlorine evolution reaction on RuO_2(110): *Ab initio* atomistic thermodynamics study – Pourbaix diagrams. *Electrochim. Acta* **120**, 460−466 (2014).

18. Pourbaix, M. *Atlas of Electrochemical Equilibria in Aqueous Solutions* (National Association of Corrosion Engineers, Houston, 1974).

19. Hansen, H. A., Rossmeisl, J. & Nørskov, J. K. Surface Pourbaix diagrams and oxygen reduction activity of Pt, Ag and Ni(111) surfaces studied by DFT. *Phys. Chem. Chem. Phys.* **10**, 3722−3730 (2008).

20. Hansen, H. A. *et al.* Electrochemical chlorine evolution at rutile oxide (110) surfaces. *Phys. Chem. Chem. Phys.* **12**, 283−290 (2010).

21. Kattel, S., Duan, Z. & Wang, G. Density functional theory study of an oxygen reduction reaction on a Pt_3Ti alloy electrocatalyst. *J. Phys. Chem. C* **117**, 7107−7113 (2013).

22. Xiao, B. B., Jiang, X. B. & Jiang, Q. Density functional theory study of oxygen reduction reaction on Pt/Pd_3Al(111) alloy electrocatalyst. *Phys. Chem. Chem. Phys.* **18**, 14234−14243 (2016).

23. Exner, K. S., Sohrabnejad-Eskan, I., Anton, J., Jacob, T. & Over, H. Full free energy diagram of an electrocatalytic reaction over a single-crystalline model electrode. *ChemElectroChem* **4**, 2902−2908 (2017).

24. Exner, K. S., Sohrabnejad-Eskan, I. & Over, H. A universal approach to determine the free energy diagram of an electrocatalytic reaction. *ACS Catal.* **8**, 1864−1879 (2018).
